# Reversible stapling of unprotected peptides *via* chemoselective methionine bis-alkylation/dealkylation†
†Electronic supplementary information (ESI) available. See DOI: 10.1039/c7sc05109c


**DOI:** 10.1039/c7sc05109c

**Published:** 2018-02-26

**Authors:** Xiaodong Shi, Rongtong Zhao, Yixiang Jiang, Hui Zhao, Yuan Tian, Yanhong Jiang, Jingxu Li, Weirong Qin, Feng Yin, Zigang Li

**Affiliations:** a Key Laboratory of Chemical Genomics , School of Chemical Biology and Biotechnology , Peking University Shenzhen Graduate School , Shenzhen , 518055 , China . Email: lizg@pkusz.edu.cn ; Email: yinfeng@pkusz.edu.cn; b Division of Life Sciences , Clarivate Analytics , Beijing , 100190 , China; c School of Life Science and Engineering , Southwest Jiaotong University , Chengdu , 611756 , China

## Abstract

We have developed a general peptide macrocyclization strategy that involves a facile and chemoselective methionine bis-alkylation/dealkylation process. This method provides a straightforward and easy approach to generate cyclic peptides with tolerances of all amino acids (including Cys), variable loop sizes, and different linkers. The Met bis-alkylation we apply in this strategy yields two additional on-tether positive charges that could assist in the cellular uptake of the peptides. Notably, the bis-alkylated peptide could be reduced to release the original peptide both *in vitro* and within cellular environments. This strategy provides an intriguing and facile traceless post-peptide-synthesis modification with enhanced cellular uptakes. Peptides constructed with this method could be utilized to zero in on various protein targets or to achieve other goals, such as drug delivery.

## Abstract 2

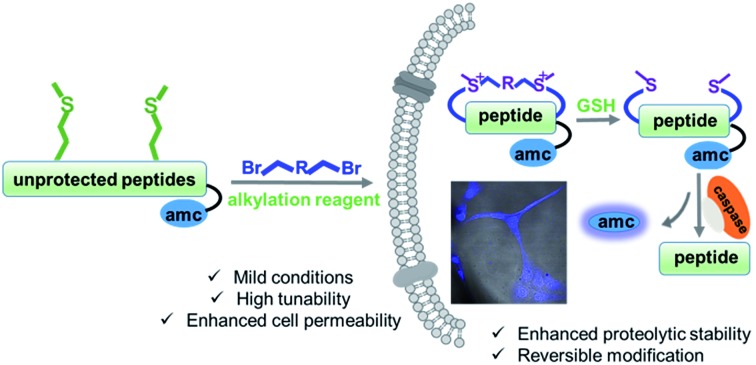
A general peptide reversible macrocyclization strategy is developed based on a facile and chemoselective methionine bis-alkylation/dealkylation process.

## Introduction

Because of their pivotal role in modulating life processes, protein–protein interactions (PPI) provide a rich space for drug development.^1^ Although peptides have been, and continue to be, broadly utilized as efficient PPI modulators, their intrinsic instability *in vivo* coupled with poor cellular uptake largely reduce their therapeutic potential.^2^ Constrained peptides, which are chemically “locked” into bioactive conformations and have improved proteolytic stability and membrane permeability, represent promising candidates for the next-generation of peptide therapeutics.^3^ Therefore, the endeavor to chemically stabilize peptides in order to attain desirable biophysical properties is an effort that has piqued the interests of both industry and academia.^4^ These efforts, in general, have involved a precisely designed tether that is chemically added to the peptides at different positions, either as a side cross tether,^5^ an N-capped nucleated moiety, or a hydrogen bond surrogate.^6^ Stabilized peptides have proven useful in targeting various intracellular PPIs, including p53/Mdm2, estrogen receptor α (ER-α), BCL-2 family proteins, hypoxia-inducible factor (HIF), Notch complexes, and so on.^7^ For the most part, the additional chemical modifications were intentionally designed at the solvent exposed face in order to avoid unwanted interactions with target proteins. However, as both proteins and peptides are flexible molecules, structural evidence indicates that the tether can contribute to the protein-peptide interactions.^8^ Contrastingly, in many cases peptides stabilized with additional tethers were found to have detrimental effects on binding with their targets, as reported by Baumach *et al.* on somatostatin receptor subtype 2 (SST2)^9^ and Mizejewski *et al.* on human alpha fetoprotein (HAFP).^10^ This effect is understandable but still problematic, and highlights the need for newer peptides that maintain the advantages of conventional constrained peptides, but avoid unwanted tether/protein interactions.

**Fig. 1:**
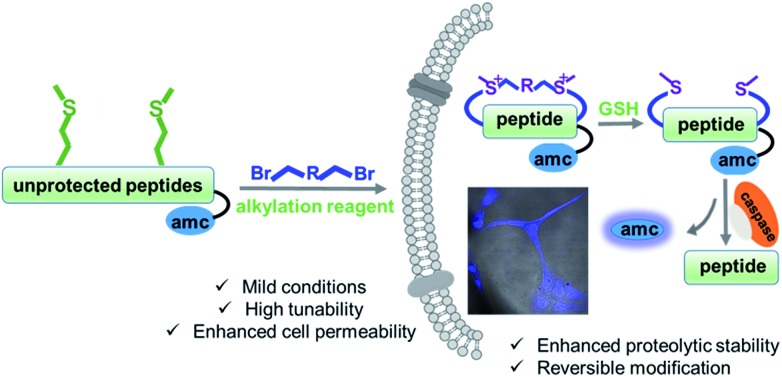
Schematic presentation of the Met bis-alkylation/dealkylation strategy for peptide macrocyclization. GSH = glutathione.

Thus, we set out to develop a post-peptide-synthesis modification, which would do just that. The enhanced serum stability and cellular uptake hallmarks of conventional peptides were preserved, while the original peptide was released upon intracellular stimulus, deflecting the potential for any unwanted interactions. The intracellular reductive cleavage of disulfide bonds was well utilized for various applications.^11^ However, disulfide bonds typically do not show a significant enhancement in uptake. Very recently, Grison *et al.* introduced a GSH reducible dibromomaleimide linker into two model peptides (BID and RNase S) that led to enhancements of helicity and proteolytic resistance.^12^ Notably, the linker could be further modified with biotin, fluorescein, and PEG azides. Deming *et al.* systematically studied the reversible alkylation of Met in linear peptides,^13^ and we reported a reversible alkylation of thioether-tethered cyclic peptides with preferable biophysical properties.^14^ Herein, we report a straightforward and traceless modification of peptides by bis-alkylation/dealkylation of Met residues that resulted in satisfying yields and excellent functional group tolerance.^15^ Peptides modified with this method showed enhanced cellular uptake, negligible cytotoxicity, and were reducible upon GSH treatment, as summarized in Fig. 1.

This convenient and triggered release strategy significantly broadens the chemical space of stabilized peptides and could have many important uses among various research areas, including therapeutic development, probing peptides, biomaterial, and drug delivery.

## Results and discussion

**Fig. 2:**
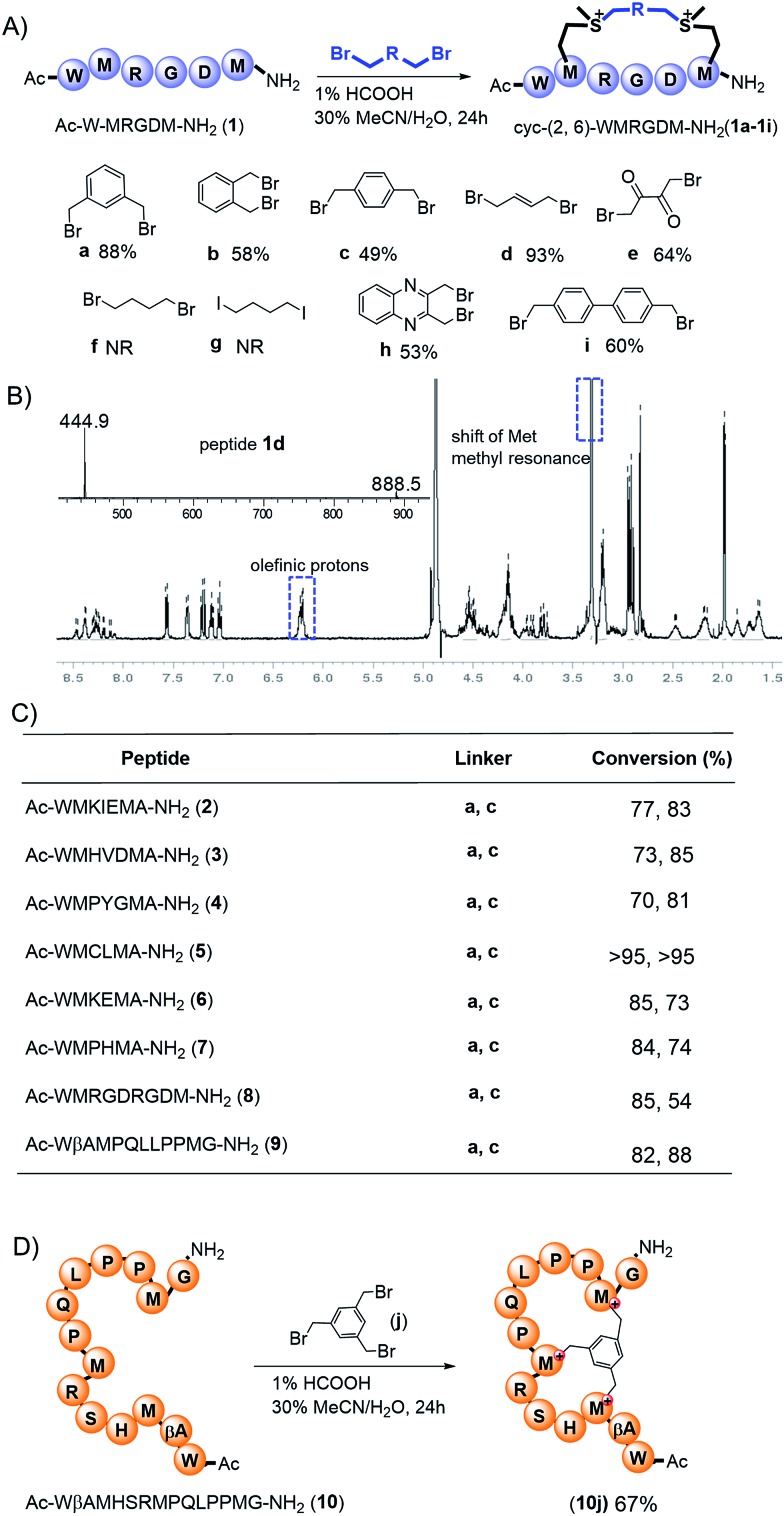
Preparation of the cyclic peptides *via* methionine alkylation. (A) Met bis-alkylation of peptide **1** with different linkers. Peptides were dissolved in a solution of 1% HCOOH MeCN/H_2_O (30 : 70, v/v) and stirred for 24 h at room temperature. The optimization process was summarized in Table S1.† NR: no reaction. (B) ESI-MS and ^1^H-NMR spectra of peptide **1d** in CD_3_OD. (C) Different peptide sequences and loop sizes cyclized by Met bis-alkylation. (D) Peptide **10** with three Mets reacted with 1,3,5-tris(bromomethyl) benzene to generate bicyclic peptide **10j**. Conversions: [desired product/(desired product + starting material)] were determined by integration of reverse-phase HPLC.

First, we utilized a model peptide with an RGD moiety, shown in Fig. 2A, to screen for suitable Met bisalkylation conditions (Table S1†). RGD was intentionally used as model due to its broad use as a cancer cell-targeting peptide as well as its bulky Arg/Asp residues, which could be used to examine the reaction's chemoselectivity and functional group tolerance.^16^ Based on our trials, this reaction could not be applied in solid phase synthesis. This may be due to the low nucleophilicity of the residue and its pseudo-dilution effect in solid phase, or the degradation of cyclized peptides in the resin cleavage cocktail (TFA/TIS/EDT/H_2_O).^17^ The modification was smoothly fulfilled in the solution phase with high conversion. Using these optimized conditions, we tested different di-alkylating linkers (Fig. 2A). The simple alkyl linkers, **f** and **g**, with significant lower electrophilicity did not react under the standard conditions. Based on HPLC-MS analysis, the other six dibromide linkers reacted smoothly with peptide **1** to generate corresponding bis-alkylation products with satisfying conversions. Linker **e** yielded a small amount of mono alkylated product (Fig. S3†). The product **1d** was confirmed by ESI-MS with the addition of 54 Da and ^1^H-NMR analysis showing a clear shift of Met methyl and olefinic protons (Fig. 2B and S1†). Peptides with different residues and loop sizes were tested for the reaction scope. Linkers **a** and **c**, which are commonly used in the peptide stapling,^5^ were used with two Met positioned at i, i+3, i+4, and i+7 respectively, and reacted smoothly with satisfying conversions, as shown in Fig. 2C. The different cyclization efficiency of peptide **5**, **6**, and **7** showed that both spacer and sequences influenced the cyclizing efficiency (Fig. 2C and S3†). Notably, peptide **5** reacted smoothly, providing peptide **5a**/**5c** with high conversion and an intact Cys after the reaction. The free thiol group on peptide **5a** was confirmed by the 5,5′-dithiobis-(2-nitrobenzoic acid) (DTNB) assay^18^ and confirmed by HPLC-MS as shown in Fig. S2.† Furthermore, this method could be easily extended to construct a bicyclic peptide. Peptide **10**, with three Mets (Fig. 2D), reacted smoothly with 1,3,5-tris(bromomethyl) benzene to generate bicyclic peptide **10j**. Based on our experimental observation, this method could provide an efficient and general approach towards generating cyclic peptides with different linkers and different loop sizes.

Unlike most of the present cyclic peptide construction strategies, our method does not require preparation of precious unnatural amino acids and laborious experimental operation. In general, peptides could be purchased from commercial sources and directly used. Additionally, peptides cleaved from the resin could be used directly for cyclization without the need for time-consuming HPLC purification.

**Fig. 3:**
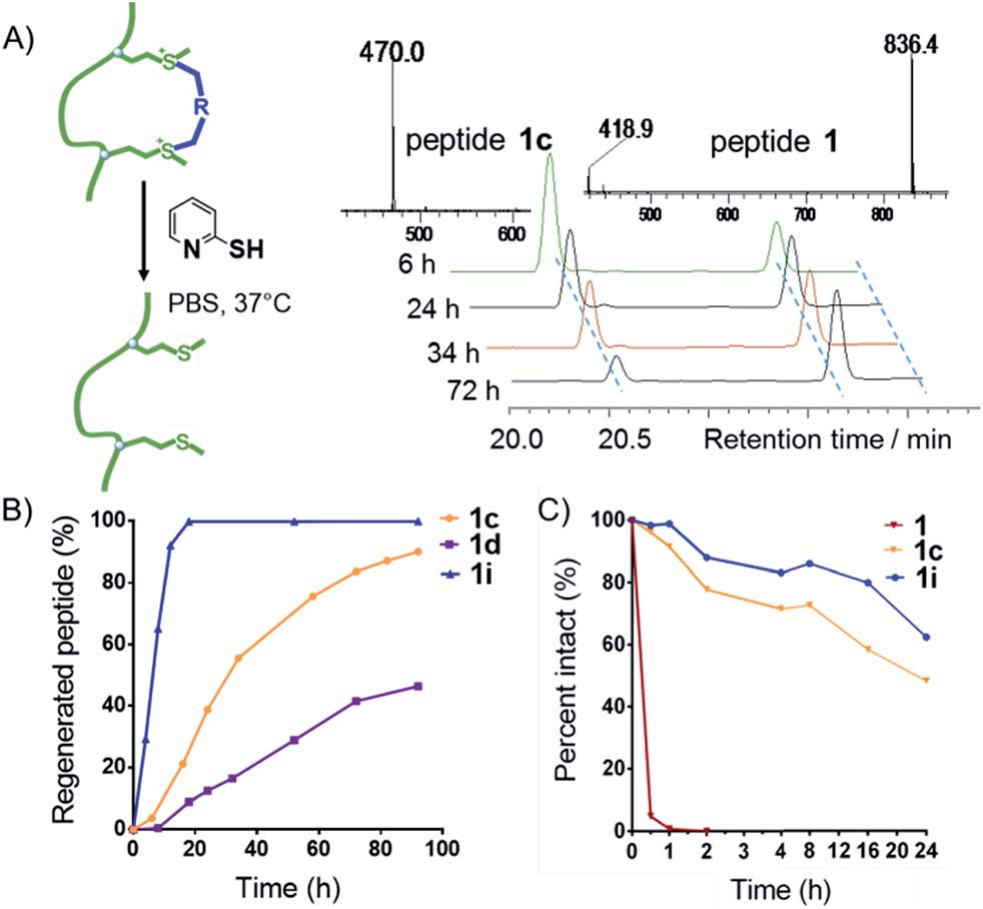
Bis-alkylated peptides are reducible and more resistant to proteolysis. (A) Dealkylation of model peptides **1c**, **1d**, and **1i** (1 mmol) in the presence of PyS (10 mmol) in PBS (pH = 7.4) at 37 °C. HPLC traces of dealkylation process of peptide **1c** at different time intervals were presented. (B) Plot of regenerated peptides *versus* time. (C) *In vitro* trypsin (1%) digestion assay. Peptide **1**, **1c**, and **1i** (1 mmol) incubated at 37 °C for different time intervals. The amounts of the peptides were examined by integration of reverse-phase HPLC.

Deming *et al.*, along with our previous research, demonstrated that sulfonium is reducible to regenerate the original peptides under reductive conditions.^13^,^14^ We tested cyclized peptides **1c**, **1d**, and **1i** under reductive conditions (Fig. 3A) and found that upon treatment with 2-mercaptopyridine (10 equiv., 10 mM) in PBS buffer (pH = 7.4), all peptides showed time-dependent regeneration of the parental peptide **1** (Fig. 3A and B). Peptide **1i** showed the quickest dealkylation, in opposition to alkylation kinetics. As shown in Fig. 3C, the bis-alkylation cyclization significantly enhanced the trypsin stability of peptides **1c** and **1i**, compared to their parental linear peptide **1**.

**Fig. 4:**
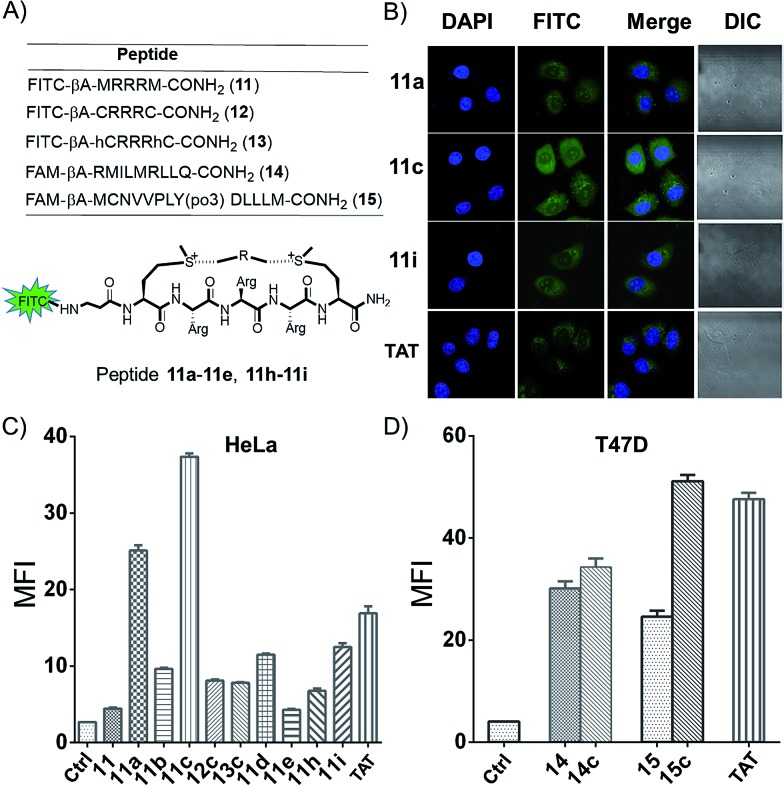
Met bis-alkylation increases the peptides' cellular uptakes. (A) A schematic presentation of peptides. (B) Confocal microscopy images of HeLa cells after treatment for 4 h with 10 μM peptides **11a**, **11c**, **11i**, and **TAT**. (C) Flow cytometry comparison of the cellular uptake efficiency of the FITC labelled peptides **11a–e**, **11h**, and **11i**, thioether cyclic peptides **12c** and **13c**, and the cell penetrating peptide **TAT** (10 μM, 4 h) in Hela cells. (D) Flow cytometry comparison of the cellular uptake efficiency of the FAM labelled peptides **14**, **14c**, and **15**, **15c** (10 μM, 4 h) in T47D cells in the medium containing 10% fetal bovine serum.

A major limitation of peptides' therapeutic application is poor cellular uptake, and peptide stabilization could provide an intriguing solution.^1^,^19^ With two additional on-tether positive charges from the sulfonium, we envisioned this modification strategy might lead to a facile and reversible approach for the generation of cell permeable peptides. In order to examine the cyclic peptide's permeability, peptide **11** FITC-βAMRRRM-NH_2_ was cyclized using linker **a–e**, **h**, and **i**, and tested in HeLa and U2OS cells using a FACS assay (Fig. 4A). As controls, we prepared peptides **12** (FITC-βA-CRRRC-NH_2_) and **13** (FITC-βA-hCRRRhC-NH_2_, hC = homo-Cys) that were cyclized using linker **c** and the cell penetrating peptide **TAT** (FITC-βA-RKKRRQRRR). According to the results of confocal microscopy imaging, the sulfonium cyclic peptides displayed a diffuse cellular distribution in HeLa cells, and the majority of the peptides were distributed in the cytoplasm and a small fraction was also detected in the nucleus (Fig. 4B). As shown in Fig. 4C and S4,† all cyclized peptides exhibited better cellular uptakes than peptide **11**, with peptide **11c** showing the best uptake. In the presence of 10% fetal bovine serum, the cellular uptake of peptide **11a**, **11c** and **11i** in HeLa cells show the same tendency as in the absence of serum at different concentrations (Fig. S5†). MTT assay show that there are no obvious cytotoxicity of these cationic peptides at 60 μM in HeLa cells even incubated for 12 h (Fig. S6†). To explore the on-tether positive charge effects, peptide **12c** and **13c** were prepared,^3^ and we observed that peptide **11c** showed a better cellular uptake than the thioether cyclic peptide. The superior cellular uptakes of peptide **11c** may be attributed to the on-tether positive charges. As peptide macrocyclization is not the ubiquitous effect for enabling intracellular access,^20^ the cellular uptakes of peptide FAM-βA-RMILMRLLQ-CONH_2_ (**14**) and FAM-βA-MCNVVPLY(po3)DLLLEM-CONH_2_ (**15**) containing different sequences cyclized by linker **c** were tested in T47D cells (Fig. 3D). The results showed that peptide **14c** showed very limited improvement of cellular uptake than its linear precursor, while peptide **15c** with a head-to-tail cyclization manner showed significantly improved cellular uptake than its linear precursor.

**Fig. 5:**
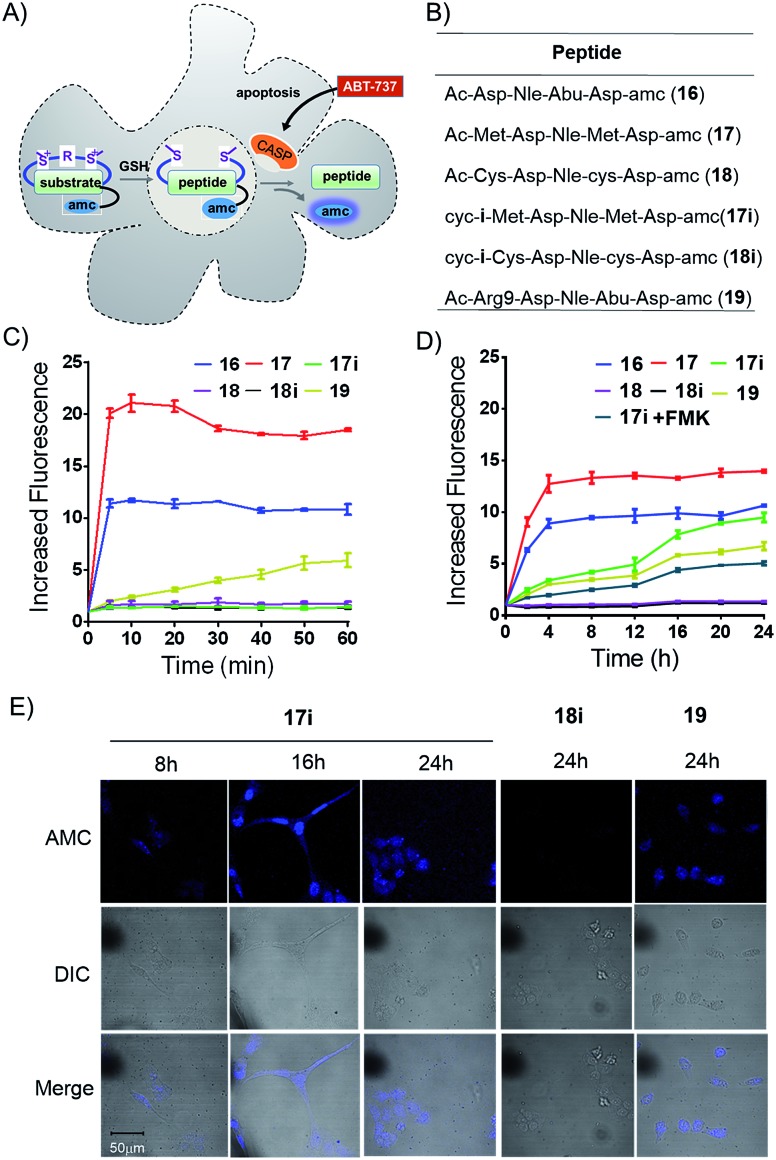
(A) Schematic illustration of the AMC release assay. (B) Peptides used for the AMC release assay. (C) AMC release efficiency of peptides **16–19** (5 μm) treated with caspase-3 protein for 1 h and monitored at 5 minute intervals. (D) Time-dependent release of AMC of peptides **16–19** (5 μm) treated with U2OS cell lysates in the absence and presence of caspase inhibitor FMK (100 μm) for 24 h. (E) Confocal microscopy images of U2OS cells after treatment with peptides **17i**, **18i** and **19** at 100 μM for 4 h, followed by incubation with GSH at 10 mM for 8, 16, or 24 h and then treatment with the apoptotic inducer ABT-737 (10 μM) for 1 h.

We prepared peptides (**16–19**) for the AMC release assay to test whether or not the tether is reducible under cellular circumstances (Fig. 5A and B). Peptide **16** was a reported substrate of caspase-3, while peptide **17** and **18** were linear control peptides based on mutations of peptide **16**. Peptide **17i** was a Met bis-alkylated peptide **17** with linker **i**, while Peptide **18i** was a Cys bis-alkylated peptide **18** with linker **i** as a negative control. Peptide **19** was peptide **16** fused with an N-terminus R9 for better cellular uptake (Fig. 5B). First, the peptides were allowed to react with caspase-3, and then monitored by an enVision multilable plate reader (*E*_x_ = 340 nm, *E*_m_ = 450 nm) based on AMC cleavage.^21^ Results from the *in vitro* caspase-3 assays clearly showed that the constrained peptide **17i** was not a suitable substrate for caspase-3 and that peptide **17**'s quick degradation suggested the addition and mutation of Met would not interfere with the enzyme's activity. Peptide **18** and **18i** showed no caspase-3 activity *in vitro*. Peptide **19** showed decreased susceptibility to caspase-3 compared with peptide **16** (Fig. 5C). Subsequently, U2OS cells were treated by the apoptosis inducer ABT-737 (10 μM) for 1 hour to induce caspase activity, and then were lysed and collected and allowed to react with different peptides (Fig. 5D). Compared with the *in vitro* kinetic test, the AMC release efficiency of peptide **17i** increased gradually during 24 h, indicating that peptide **17i** was defalcated to the linear peptide and recognized by caspses-3. As expected, when using the U2OS cell lysate pretreated with the pan caspase inhibitor Z-VAD–(OMe)–CH_2_F (FMK), peptide **17i**'s caspase-3 activity was reduced significantly (Fig. 5D). However, peptide **18i**, which was cyclized by an irreversible method, showed no susceptibility to caspase-3. The reducible properties of peptide **17i** was also observed in cell by the AMC release assay (Fig. S9†). After incubation for 12 hours, **17i** show higher caspase-3 activity than the linear peptide **16**, while lower than **19**, suggesting that the reduction of peptide **17i** is a slow process in cell. The confocal microscopy assays were carried out to further confirm the reversible nature of the bis-alkylation of Met. U2OS cells were treated with peptides for 4 hours to allow adequate cellular uptake, then washed to remove any of the peptides in solution, and finally incubated in fresh medium with additional 10 mM GSH to accelerate the reduction procedure. At different time intervals, the cells were treated with ABT-737 (10 μM, 1 h) to induce caspase activity, and then fixed (Fig. 5A). As shown in Fig. 5E, under the 405 nm excitation, peptide **17i** was lighted gradually. In the presence of 10% fetal bovine serum, peptide **17i** could also be lighted at 50 μM while with a weaker fluorescence intensity which likely due to the slow reduction process in the cellular environment at a relative lower concentration (Fig. S7A†). Peptide **19** was lighted at an earlier time and also exhibited a membrane lysis effect (Fig. S7B and S8†). Lactate dehydrogenase (LDH) release assay of the sulfonium peptide **17i** and **18i** showed negligible membrane toxicity at 160 μM (Fig. S8†).

No fluorescent signals were observed in cells treated by peptide **18i** following 24 hours. Taken together, we concluded that, under the adopted conditions, the sulfonium bond cyclized peptide **17i** was dealkylated and recognized by the caspase.

## Conclusions

In conclusion, we have herein reported a chemoselective bis-alkylation/dealkylation of Met that provides a facile and reversible peptide stapling strategy. This method yields satisfying functional group tolerance and conversion yet only requires simple operation on commercially available peptides. Because two additional positive charges are added onto the tether *via* sulfonium bond formation, the resulting cyclic peptides show better cellular uptakes and enzymatic stability than their linear parental peptides. This process is straightforward, mild, functional-group-tolerant, and high-yielding, and one could easily design suitable modifications on the tether or on the peptides.^22^ Numerous potential applications could be achieved based on this traceless modification strategy,^23^ and investigations for utilizing this strategy to construct PPI ligands or develop drug delivery vectors are currently underway in our laboratory and will be reported in due course. As a proof of concept study, this method provides an intriguing alternative to those that are presently utilized for the stabilization of peptides. We are at this time investigating new modifications of both Met and linkers in our laboratory. We believe further development of this strategy could lead to standardization of a facile peptide modification protocol that could be easily utilized by scientists in both the biological or material research fields.

## Conflicts of interest

There are no conflicts of interest to declare.

## Supplementary Material

Supplementary information
