# Supplementary material for: Reversible stapling of unprotected peptides via chemoselective methionine bis-alkylation/dealkylation
Source: Chem Sci. 2018 Feb 26;9(12):3227–32. doi: 10.1039/c7sc05109c (PMC5931191; doi:10.1039/c7sc05109c)
Supplement: Supplementary file 1 [file SC-009-C7SC05109C-s001.pdf]

Electronic Supplementary Material (ESI) for Chemical Science.
This journal is © The Royal Society of Chemistry 2018

Supporting Information

Reversibly Stapling of Unprotected Peptides via Chemoselective Methionine Bis-alkylation/Dealkylation

Xiaodong Shi,[a] Rongtong Zhao,[a] Yixiang Jiang,[a] Hui Zhao,[b] Yuan Tian,[c] Yanhong Jiang,[a] Jingxu Li,[a] Weirong Qin,[a] Feng Yin*[a] and Zigang Li*[a]

a. Key Laboratory of Chemical Genomics, School of Chemical Biology and Biotechnology, Peking University Shenzhen Graduate School, Shenzhen, P. R. China. E-mail: lizg@pkusz.edu.cn, yinfeng@pkusz.edu.cn.

b. Division of Life Sciences, Clarivate Analytics, Beijing, 100190 (China).

c. School of Life Science and Engineering, Southwest Jiaotong University, Chengdu, 611756 (China)

General information

1. Abbreviations

Fmoc, 9-fluorenylmethyloxycarbonyl; HCTU, 2-(1H-6-chlorobenzotriazol-1-yl)-1,1,3,3-tetramethyl uranium hexafluorophosphate; HATU, 2-(7-Aza-1H-benzotriazole-1-yl)-1,1,3,3-tetramethyluronium hexafluorophosphate ; DIPEA, diisopropylethylamine; DMF, dimethylformamide; DCM, dichloromethane; TFA, trifluoroacetic acid; TIS, triisopropylsilane; Et_2O, diethyl ether; LC-MS, liquid chromatography–mass spectrometry; NMR, nuclear magnetic resonance; HPLC, high-performance liquid chromatography; ESI-MS, electrospray ionization mass spectrometry; RP-HPLC, reserved-phase high performance liquid chromatography; RT, room temperature; SPPS, solid-phase peptide synthesis.

2. Materials

The building block used for solid phase peptide synthesis were purchased from GL Biochem. The solvents and reagents used for solid phase peptide synthesis were purchased from commercial suppliers including GL Biochem (Shanghai) Ltd., Shanghai Hanhong Chemical Co., J&K Co. Ltd., Shenzhen Tenglong Logistics Co. or Energy Chemical Co. and were used without further purification unless otherwise stated.

3. Peptide Synthesis and Characterization

All peptides were synthesized by manual Fmoc-based solid-phase synthesis. 1) Swelling: Rink Amide MBHA resin (loading capacity: 0.34 mmol/g) (GL Biochem Ltd.) was put into the tube; DMF was added and the resin was bubbled with N_2 for 10 min. 2) Deprotection: Fmoc deprotection was performed with morpholine (50% in DMF) for 30min × 2. 3) Washing: The resin was washed sequentially with DCM and DMF (5 times). 4) coupling: Fmoc-protected amino acids (5.0 equiv according to initial loading of the resin) and HCTU (4.9 equiv) were dissolved in DMF, followed by DIPEA (10.0 equiv)，and the mixture was pre-activated for 1 min and added to the resin for 1-2 h, then the resin was washed with DCM and NMP (5 times). Repeat the 3 steps until all the amino acid residues are assembled. Unnatural amino acids was used as 3 equivalents. 5) N-termianl acetylation: upon completion of peptide assembly, peptides were N-terminally acetylated with a solution of acetic anhydride and DIPEA in NMP (1:4: 20 in volume) for 30min × 2. Peptides were cleaved from the resin with a mixture of TFA/H2O/EDT/TIS (94:2.5:2.5:1) for 2 h and concentrated under a stream of nitrogen. The crude peptides were then precipitated with Hexane/Et_2O (1:2 in volume) at 4°C, isolated by centrifugation then dissolved in water/acetonitrile (3:1 in volume), purified by HPLC and analyzed by LC-MS. Acetylated peptides were quantified by their UV absorbance at 280 nm. Peptides **12**, **13**, **14**, **15** conjugated with amc (7-amino-4-methylcoumarin) were purchased from GL Biochem.

4. HPLC and Mass spectrometry

Peptides were analyzed and purified by HPLC (SHIMAZU Prominence LC-20AT) using a C18 analytic column (Agilent

ZORBAX SB-Aq, 4.6 × 250 mm, 5 μm, flow rate 1.0 mL/min) and a C18 semi-preparative column (Agilent Eclipse XDB-C18, 9.4 × 250 mm, 5 μm, flow rate 5 mL/min). H_2O (containing 0.1% TFA) and pure acetonitrile were used as solvents in linear gradient mixtures. LC-MS spectra were carried out on SHIMAZU LC-MS 8030 (ESI-MS) in positive ion mode.

Experimental section

1. Preparation of alkylated sulfonium peptides[1]

Linear peptide was cleaved from the resin and precipitated with Hexane/Et_2O (1:1 in volume), then dried in vacuum. Dissolve it in CH_3CN/H_2O (1:3 in volume) to reach a concentration of 10mM (based on the loading of the resin) followed by added with 1% (in volume) formic acid to adjust the pH of the solution to about 3. The alkylated reagent (1.2 equiv) were dissolved by a small amount of DMF and the two solution was mixed and stirred at room temperature for 24h. During the course of experiment, turbid solution would become pellucid. Then the solution was purified by reversed phase HPLC immediately.

2. Regeneration of the linear peptide by dealkyation of the cyclic peptide 1c, 1d and **1i.**[1]

Cyclic peptide (1mmol) was dissolved in 10mM nucleophile (2-mercaptopyridine) in PBS (pH=7.4) and incubated at 37℃, at different time points, an aliquot of each reaction solution was removed and monitored by LC-MS. The cyclic peptide can be gradually regenerated to the linear peptide.

3. Trypsin stability

Peptide **1**, **1c** and **1i** were prepared at a concentration of 1 mM in PBS (pH=7.4). To 100 μL of each solution 1 μL trypsin (1 mg/mL) was added and incubated at 37°C. At different time interval, the solution was mixed with 12% trichloroacetic acid (H_2O/CH_3CN: 2:1) to inactivate the trypsin. After 10000rpm centrifuge to remove the inactive trypsin, the peptide remaining was examined by LC/MS with a grace smart C18 250× 4.6mm column, using a 4% per minute linear gradient from 10% to 70% acetonitrile over 15min. The amount of starting material left in each sample was quantified by LC/MS-based peak detection at 254nm.

4. Flow Cytometry Analysis

Cell Culture. HeLa cells were maintained in medium consisting of DMEM, 10% fetal bovine serum (FBS) and 1% penicillin/streptomycin. U2OS cells were maintained in medium consisting of Micro-5A, 10% FBS and 1% penicillin/streptomycin. T47D cells were cultured in RPMI1640, 10% FBS and supplemented with 1% (v/v) antibiotics penicillin/streptomycin.

Hela or U2OS cells were seeded overnight in 12-well plates, and then treated with FITC-labelled peptides **11a**-**11e**, **11h**-**11i** and peptide **12c** -**13c** (10μM) with or not 10% FBS in the medium for 4 hr at 37°C. T47D cells were seeded in 12-well plates overnight, and then treated with FAM-labelled peptides **14/14c** and **15/15c** (10μM) in medium containing 10% FBS for 4hr at 37°C. After washing with media, the cells were exposed to trypsin (0.25%; Gibco) digestion (2 min, 37°C), washed with PBS, and resuspended in PBS. Cellular fluorescence was analyzed using a BD FACS Calibur flow cytometer (Becton Dickinson) and CellQuest Pro (or CFlow plus).

5. Confocal Microscopy.

To detect peptide internalization, Hela cells were cultured with DMEM with 10% FBS (v/v) in imaging dishes (50000 cells/well), Peptide were first dissolved in DMSO to make a stock solution and then added to cells to a final

concentration of 10 μM in FBS free medium and incubated with the cells for 4 hours at 37°C. After that, the medium was removed and the cells were gently washed 2 times with PBS and then fixed with 4% paraformaldehyde (Alfa Aesar, MA) in PBS for 10 minutes. The cells were again washed 2 times with PBS and stained with 1μg/ml 4', 6-diamidino-2-phenylindole (DAPI) (Invitrogen, CA) in PBS for 5 minutes. Images of peptide localization in cells were taken on PerkinElmer confocal microscopy under the same parameters and processed using Volocity software package (Zeiss Imaging).

6. Cell Viability by MTT assay.

100μL of ~ 10000 cells/well suspension was placed in each well of the 96 well culture plate and allowed to grow in DMEM supplemented with 10% FBS overnight. Then the cells were incubated with serial dilution of peptides at 37℃ with 10% FBS for 12 h and then 20 μL of MTT reagent was added and incubated at 37℃ for 4 h. The absorbance of formazan product was measured at 494 nm by a microplate reader (Perkin Elmer, Envision, 2104 Multilabel Reader). Cells without peptide were treated as control.

7. LDH release assay.

LDH release was performed by using Cytotoxicity LDH Assay Kit-WST®. Briefly, ~10000 cells suspension was added to each well of the 96 well plate and allowed to grow overnight. The cells were then incubated with the fresh medium containing serial dilution of peptides at 37 °C for 4h. Lysis Buffer was added as positive control at 37 °C for 30min. After that, 50μL of the incubation medium were taken out to another 96 well plate followed the addition of 50μL Working Solution and incubated for 30min at room temperature. Then 25μL Stop Solution was added into the wells. The absorbance at 490 nm was measured by a microplate reader (Perkin Elmer, Envision, 2104 Multilabel Reader). The LDH release activity was calculated by $(LDH\%_{samples} - LDH\%_{blank}) / (LDH\%_{positive\ control} - LDH\%_{blank})$.

8. AMC release Assays. [2]

In *vitro* Caspase-3 assay:

Recombinant Human Caspase-3 protein (RnD SYSTEMS) was first diluted to 0.4ng/μL by the reaction buffer (25 mM HEPES, pH 7.4, CHAPS, and 10 mM dithiothreitol) for 30 min at 37℃. AMC conjugated peptides **16**-**19** (50 μL, 5μM) were added into the wells on a 96-well plate. Then the caspase-3 protein (50μL, 0.4ng/μL) was separately added to the half of the wells to initiate the reactions, while 50 μl of reaction buffer was added to the remaining peptide containing wells as control, and the plate was monitored at 5 minutes intervals on a EnVision Multilabel Plate Reader (Ex = 340 nm, Em = 450 nm). The difference in fluorescence yields between the experiment group and the control group was plotted as a function of time to represent the caspase-3 activities. All experiments were performed in triplicates with consistent results.

Cell lysate assay:

U2OS cells were cultured in 12 well plates (~$5X10^6$ cells/well) and pretreated with ABT-737(10 μM, 1h) or PBS (as blank control) to induce the activity of caspase-3. Then cells were digested by trypsin, washed with PBS, counted and collected by centrifugation. Consequently, resuspended the induced or non-induced cells in cell lysis buffer (BioVision-caspase-3/cpp32 fluorometric assay kit) with equal density and incubated for 10 minutes on ice. Then added 50μl of the cell lysate, 50μl 2 x reaction buffer (BioVision-caspase-3/cpp32 fluorometric assay kit) and peptides **16**-**19** stock solution (final concentration of 5 μM) into a 96-well plate. Besides, an induced well pretreated with the caspase pan-inhibitor FMK (100 μM, 1 h) was designed as a control for examining that the AMC release was caspase mediated. For each peptide, after deducted the background of the blank well, the fluorescent signals of different wells with induced cell lysate or non-induced cell lysate at different times was detected to represent the caspase-3 activities. All

experiments were performed in triplicates with consistent results.

In *vivo* Caspase-3 assay:

100μl U2OS cells were cultured in 96 well plate (~5000 cells / well) overnight, then 0.1 μl of ABT-737 stock solution (10 mM, 1 h) was added into half of the wells to induce apoptosis. After that, peptide **16**, **17i**, **18i**, and **19** stock solution (final concentration of 5 μM) were added into the cells. At different time points, the fluorescence of the wells treated with or not ABT-737 were monitored by the EnVision Multilabel Plate Reader (Ex = 340 nm, Em = 450 nm). The difference in fluorescence yields between ABT-737 treated and untreated cells was plotted as a function of time to represent the caspase-3 activities in live cells.

Confocal Microscopy

U2OS cells were cultured in 24 well plates with imaging dishes (50000 cells/well) and then treated with peptide **17i**, **18i** and **19** (100μM) in FBS free medium for 4h. Then the medium was removed and the cells were cultured with a fresh medium containing 10mM GSH for 8h, 16h, or 24h. The release efficiency of AMC was monitored using fluorescence microscopy with pseudo-colored in blue as described.

Table S1. Conversion of peptide **1** to peptide **1a** in different solvents at different time intervals.

	Conversion (%)	
	12h	24h
Solid phase	NR	
H_2O/MeCN (30%)	47	88
DMF	19	36

Table S2. Peptide sequences and observed masses following HPLC purification. Calculated and Found m/z are presented as $[M+H]^+$ / $[M/2+H]^+$/ $[M/3+H]^+$/ $[M/4+H]^+$.

Peptide	Sequence	Calculated mass	Observed mass
**1**	Ac-WMRGDM-NH_2	835.3	836.4/418.9
**1a**	Ac-W-(cyclo-**a**)-MRGDM-NH_2	940.4	469.9
**1b**	Ac-W-(cyclo-**b**)-MRGDM-NH_2	940.4	470.0
**1c**	Ac-W-(cyclo-**c**)-MRGDM-NH_2	940.4	470.0
**1d**	Ac-W-(cyclo-**d**)-MRGDM-NH_2	890.4	444.9
**1e**	Ac-W-(cyclo-**e**)-MRGDM-NH_2	919.4	459.9
**1h**	Ac-W-(cyclo-**h**)-MRGDM-NH_2	991.4	495.9
**1i**	Ac-W-(cyclo-**i**)-MRGDM-NH_2	1016.4	508.0
**2c**	Ac-W-(cyclo-**c**)-MKIEMA-NH_2	1053.5	526.5
**3c**	Ac-W-(cyclo-**c**)-MHVDMA-NH_2	1034.4	516.9
**4c**	Ac-W-(cyclo-**c**)-MPYGMA-NH_2	1000.4	499.9
**5c**	Ac-W-(cyclo-**c**)-MCLMA-NH_2	899.4	449.5
**6c**	Ac-W-(cyclo-**c**)-MKEMA-NH_2	939.4	470.0
**7c**	Ac-W-(cyclo-**c**)-MPHMA-NH_2	916.4	458.4
**8c**	Ac-W-(cyclo-**c**)-MRGDRGDM-NH_2	1268.5	634.0/423.2
**9c**	Ac-W-(cyclo-**c**)- βAMPQLPPMG-NH_2	1272.6	636.1
**10j**	Ac-W-(bicyclo)-βAMHSRMPQLPPMG-NH_2	1798.8	599.0/449.5
**11**	FITC-βA-MRRRM-NH_2	1208.5	605.0/403.9/303.2

**11a**	FITC-βA-(cyclo-**a**)-MRRRM-NH_2	1313.5	656.0/437.9
**11b**	FITC-βA-(cyclo-**b**)-MRRRM-NH_2	1313.5	656.1/437.8
**11c**	FITC-βA-(cyclo-**c**)-MRRRM-NH_2	1313.5	656.1/437.8
**12c**	FITC-βA-(cyclo-**c**)-CRRRC-NH_2	1255.5	628.0/419.2
**13c**	FITC-βA-(cyclo-**c**)-hCRRRhC-NH_2	1283.5	642.0/428.5
**11d**	FITC-βA-(cyclo-**d**)-MRRRM-NH_2	1263.5	631.1/421.2/316.2
**11e**	FITC-βA-(cyclo-**e**)-MRRRM-NH_2	1293.5	646.0/431.2/323.7
**11h**	FITC-βA-(cyclo-**h**)-MRRRM-NH_2	1356.6	682.1/455.3
**11i**	FITC-βA -(cyclo-**i**)-MRRRM-NH_2	1389.6	463.2/347.8
**14c**	FAM-βA-RMILMRLLQ-$CONH_2$	1706.9	852.8/568.9/427.1
**15c**	FAM-βA-MCNVVPLY$_{(po_3)}$DLLLEM-$CONH_2$	2264.9	1132.4/755.4/567.0
**TAT**	FITC-βA-RKKRRQRRR-NH_2	1798.9	600.6/450.9
**16**	Ac-Asp-Nle-Abu-Asp-AMC	645.3	645.4
**17**	Ac-Met-Asp-Nle-Met-Asp-AMC	822.3	822.5
**18**	Ac-Cys-Asp-Nle-cys(d)-Asp-AMC	766.2	766.3
**17i**	cyclo-**i**-Met-Asp-Nle-Met-Asp-AMC	1001.4	501.5
**18i**	cyclo-**i**-Cys-Asp-Nle-cys(d)-Asp-AMC	944.3	945.5
**19**	Ac-R_9-Asp-Nle-Abu-Asp-AMC	2050.2	1026.6/6844.7

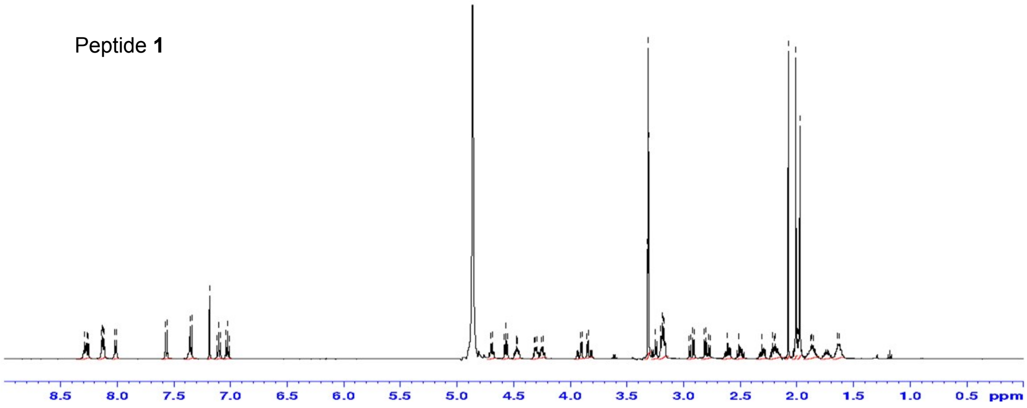

Figure S1. ^{1}H-NMR NMR spectra of peptide **1** on Bruker Avance-III 500MHz spectrometer in CD_3OD.

Figure S2. The yellow solution generated by peptide **5a** react with DTNB in the PBS buffer (pH=8.0, 4mg/ml) was examined by HPLC-MS, and the product of peptide **5a** addition of thio-nitrobenzoic acid (TNB) was detected.

Crude HPLC separation spectra of peptide Ac-W-(cyclo-**a**)-MRGDM-NH_2 (**1a**)

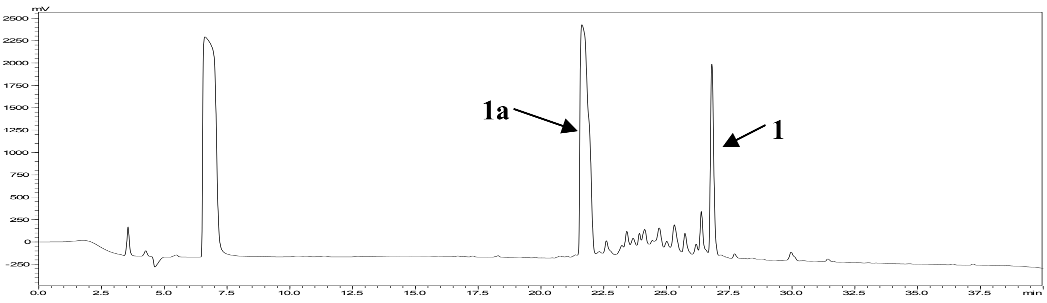

Crude HPLC separation spectra of peptide Ac-W-(cyclo-**b**)-MRGDM-NH_2 (**1b**)

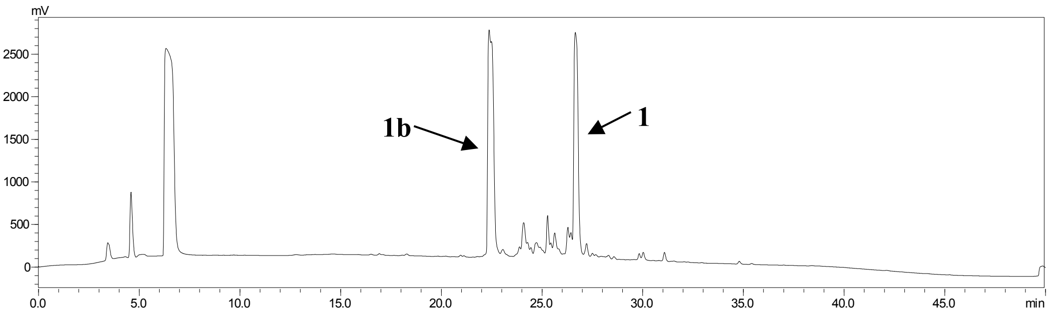

Crude HPLC separation spectra of peptide Ac-W-(cyclo-**c**)-MRGDM-NH_2 (**1c**)

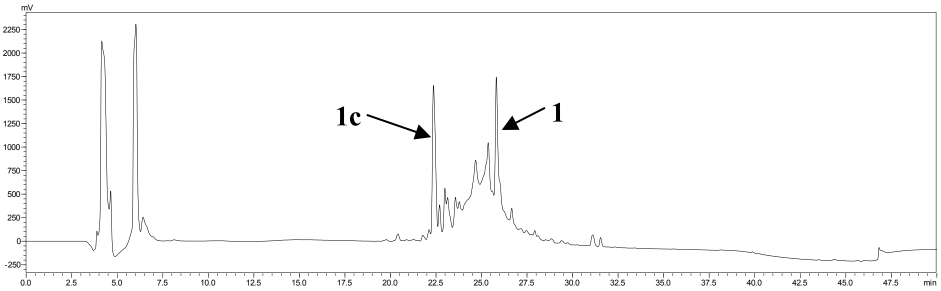

Crude HPLC separation spectra of peptide Ac-W-(cyclo-**d**)-MRGDM-NH_2 (**1d**)

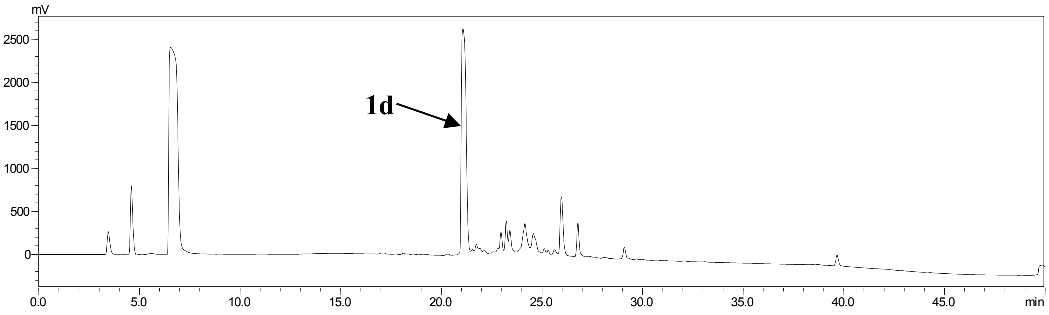

Crude HPLC separation spectra of peptide Ac-W-(cyclo-**c**)-MRGDM-NH_2 (**1e**)

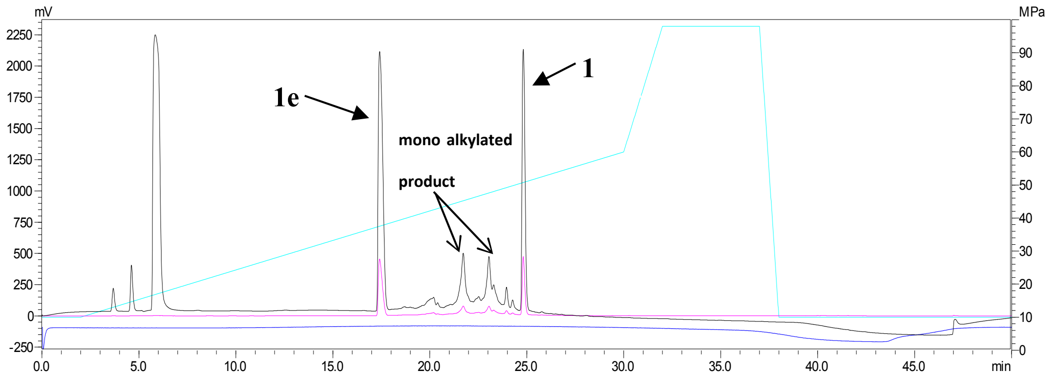

Crude HPLC separation spectra of peptide Ac-W-(cyclo-**c**)-MRGDM-NH_2 (**1h**)

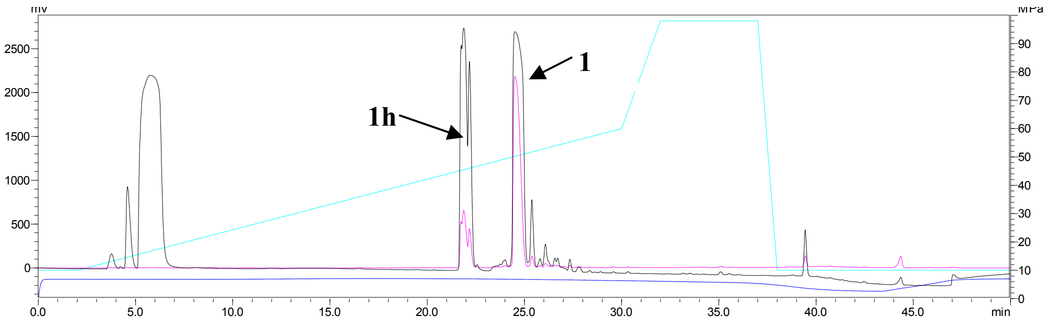

Crude HPLC separation spectra of peptide Ac-W-(cyclo-**i**)-MRGDM-NH_2 (**1i**)

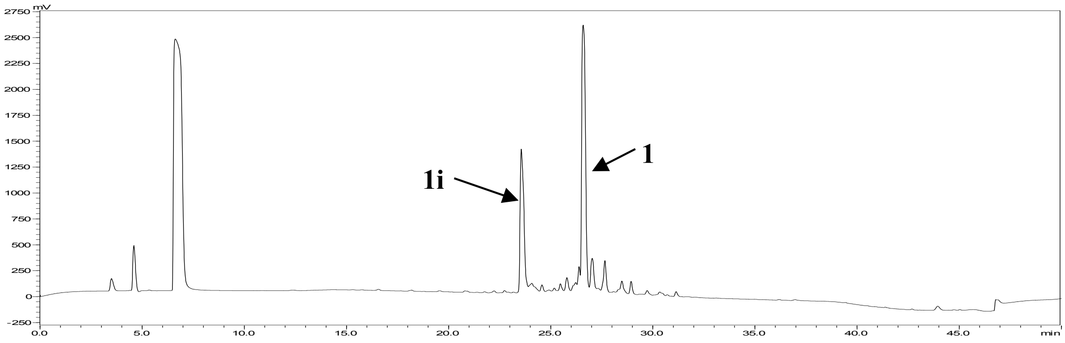

Crude HPLC separation spectra of peptide Ac-(cyclo-**a**)-WMCLMA-NH_2 (**5a**)

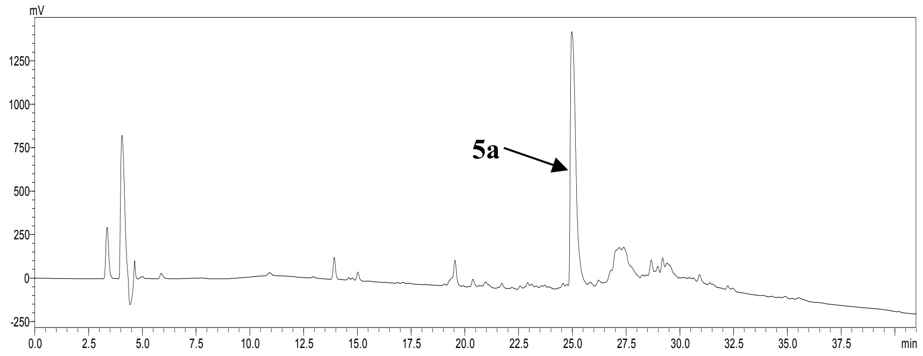

Crude HPLC separation spectra of peptide Ac-(cyclo-**a**)-WMKEMA-NH_2 (**6a**)

6a

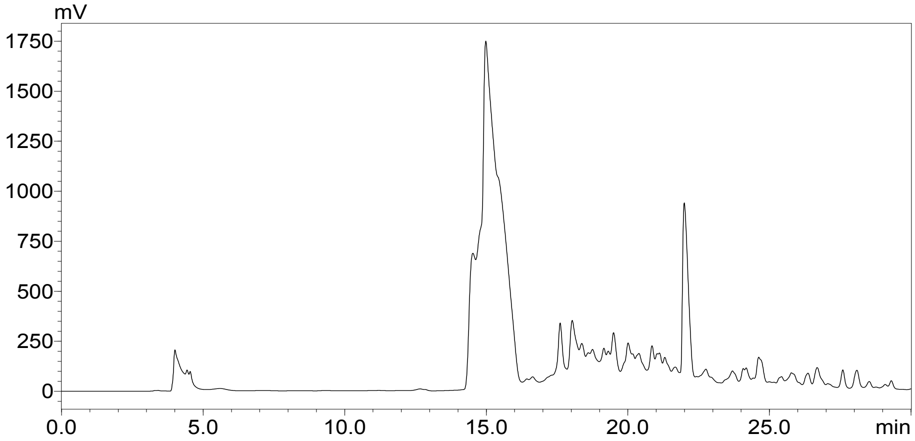

Crude HPLC separation spectra of peptide Ac-(cyclo-**a**)-WMPHMA-NH_2 (**7a**)

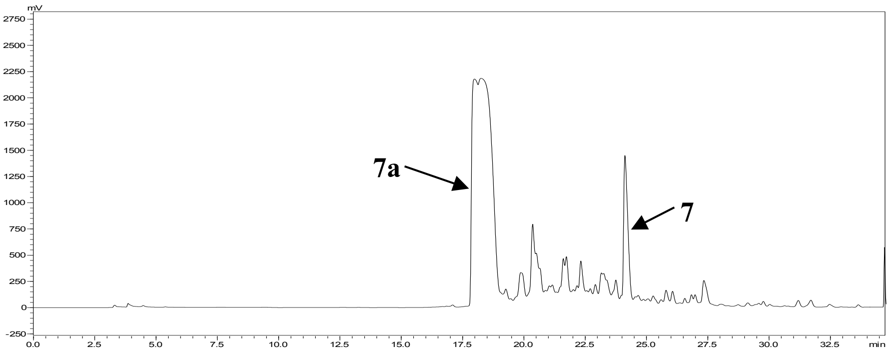

Figure S3. Crude HPLC separation spectra of peptide **1a**-**1i, 5a**-**7a**. (Column: C18 analytic column - Agilent ZORBAX SB-Aq, 4.6 × 250 mm, 5 μm.Flow rate: 1.0 mL/min. Gradient: 10% B over 2min, 10–60% (vol/vol) B over 28 min, 70%-98% over 2 min; 98% over 5min).

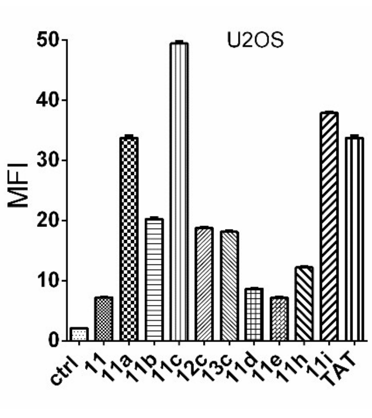

Figure S4. Flow cytometry comparison of the cellular uptake efficiency of the FITC labelled peptides **11a**-**e, 11h**, and **11i**, thioether cyclic peptides **12c** and **13c,** and the cell penetrating peptide **TAT** (10μM, 4h) in U2OS cells.

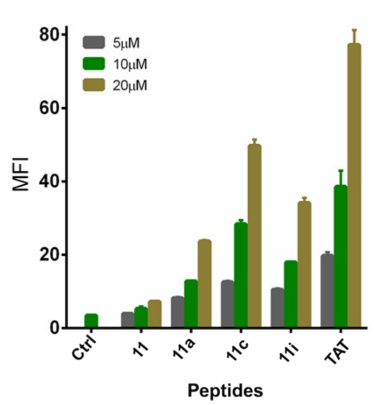

Figure S5. Flow cytometry comparison of the cellular uptake efficiency of the peptides **11**, **11a**, **11c**, **11i**, and the cell penetrating peptide **TAT** in HeLa cells in the presence of 10% fetal bovine serum at a range of concentrations (5μM, 10μM, 20μM).

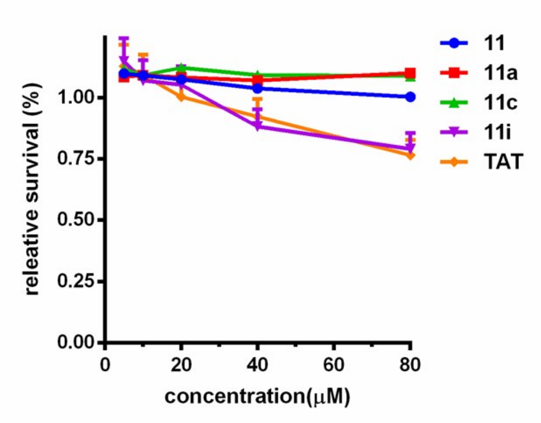

Figure S6. Relative survival of HeLa cells incubated for 12 h in the medium containing 10% FBS with the peptide samples at different concentrations (80 μM, 40 μM, 20 μM, 10 μM, 5μM,).

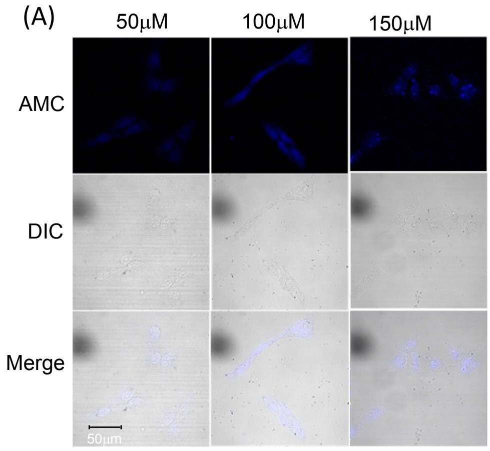

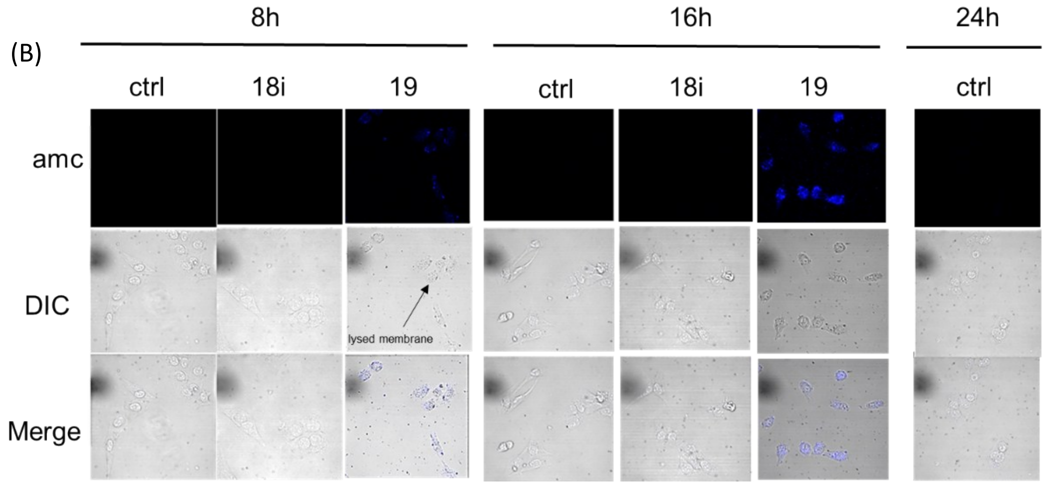

Figure S7. (A) Confocal microscopy images of U2OS cells after treatment with peptides **17i** at 50 μM, 100 μM and 150 μM for 4 h in the presence of 10% fetal bovine serum, followed by incubation with GSH (10 mM) for 16 h and then treatment with the apoptotic inducer ABT-737 (10 μM) for 1 h. (B) Confocal microscopy images of U2OS cells after treatment for 4 h with 100 μM peptides **18i** and **19** and then incubation with 10mM GSH for 8h, 16h or 24h followed by treated with the apoptotic inducer ABT-737 (10μM) for 1h.

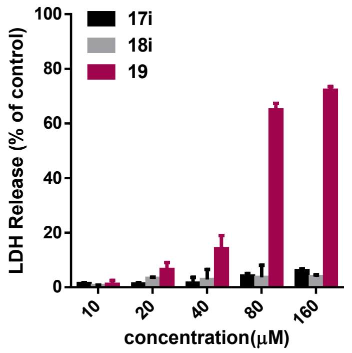

Figure S8. LDH release from U2OS cells treated with the peptides in the presence of 10% serum for 4 h at different concentrations.

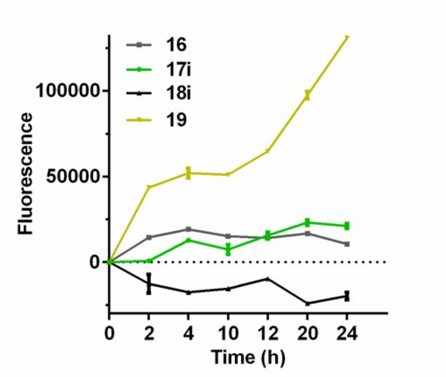

Figure S9. U2OS cells were seeded in a 96 well plate and pretreated with the apoptosis inducer ABT-737 (10 μM, 1h) or not as control. Then peptides **16**-**19** (5 μM) were added into the cells and the amount of AMC released was monitored over time (0-24h).

LC-MS spectra of peptides used in the manuscript.

Ac-WMRGDM-NH_2 (**1**)

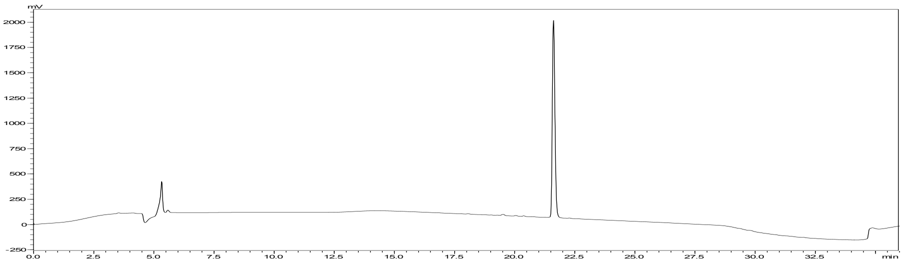

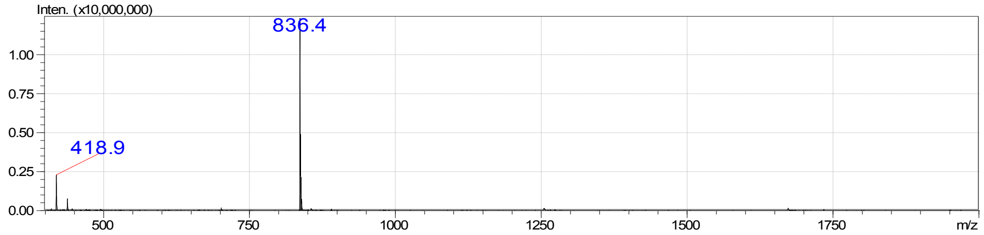

Ac-W-(cyclo-**a**)-MRGDM-NH_2 (**1a**)

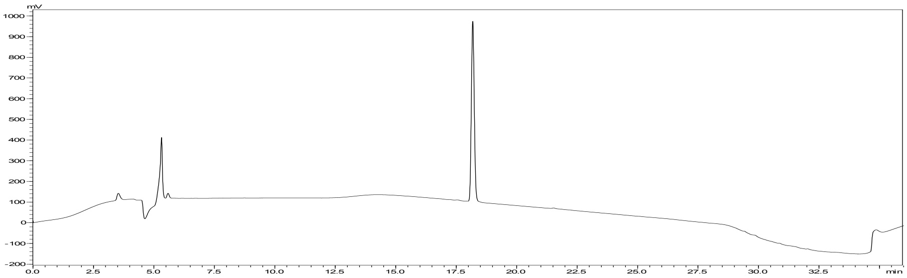

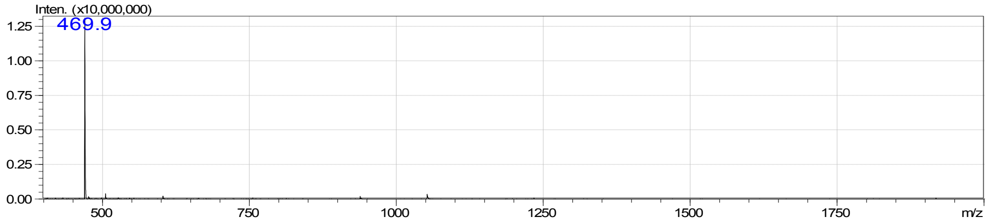

Ac-W-(cyclo-**b**)-MRGDM-NH_2 (**1b**)

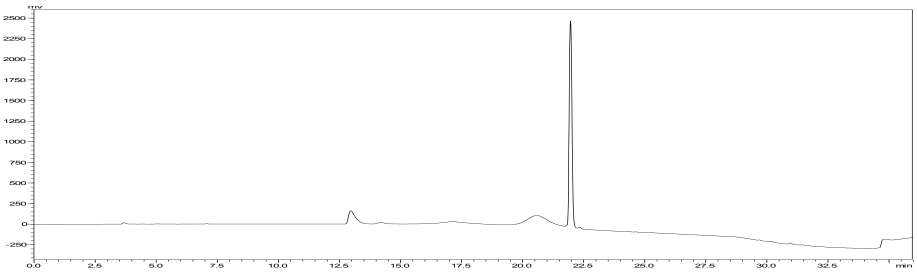

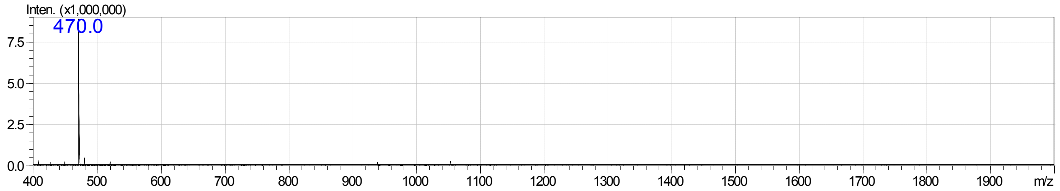

Ac-W-(cyclo-**c**)-MRGDM-NH_2 (**1c**)

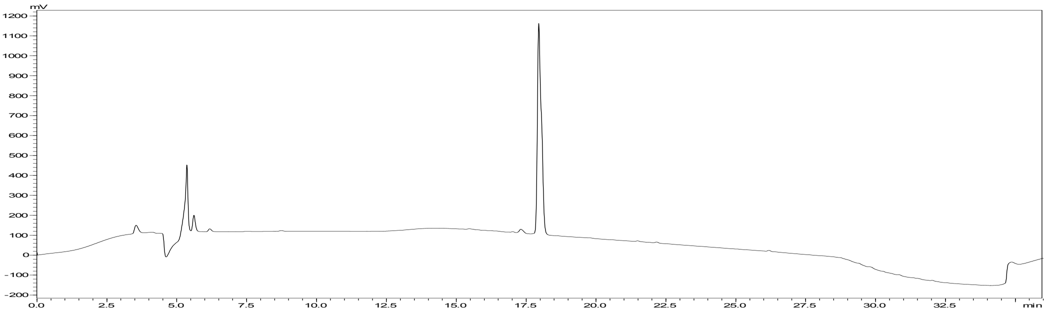

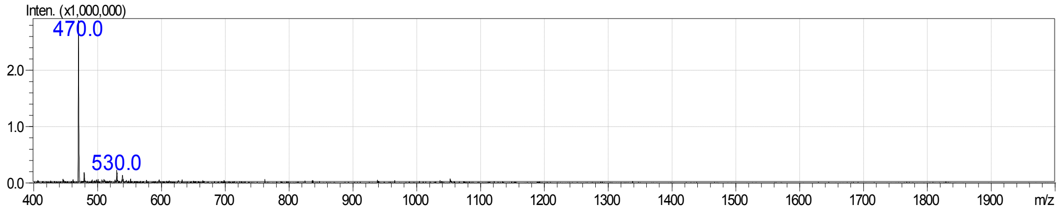

Ac-W-(cyclo-**d**)-MRGDM-NH_2 (**1d**)

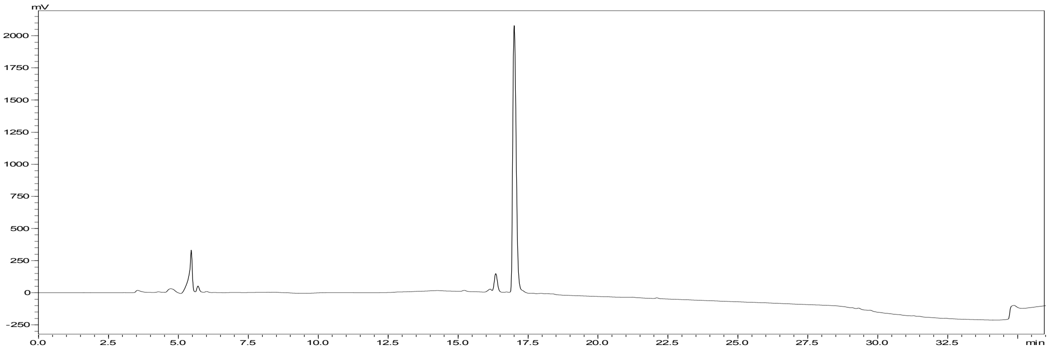

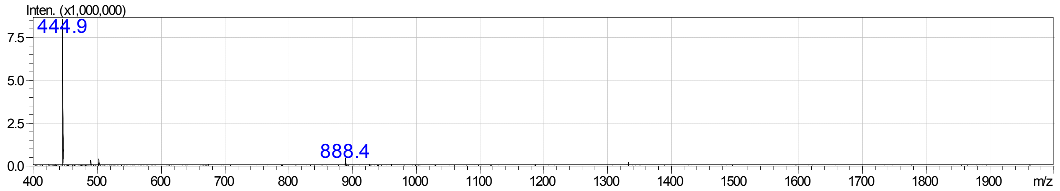

Ac-W-(cyclo-**e**)-MRGDM-NH_2 (**1e**)

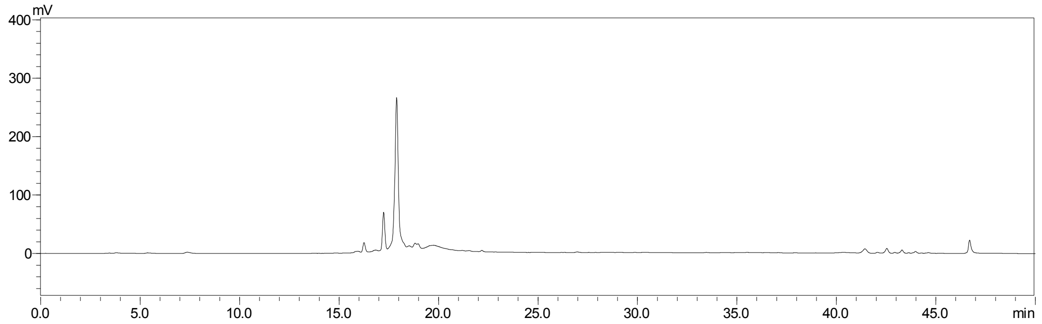

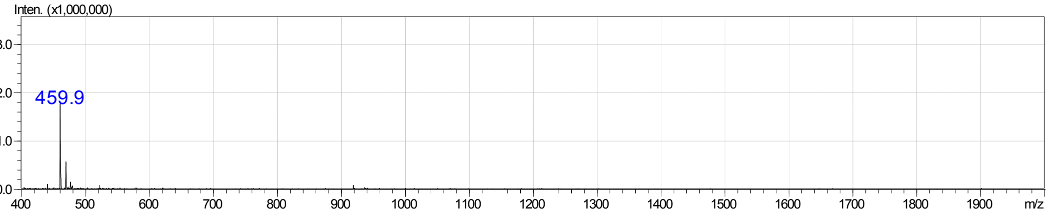

Ac-W-(cyclo-**h**)-MRGDM-NH_2 (**1h**)

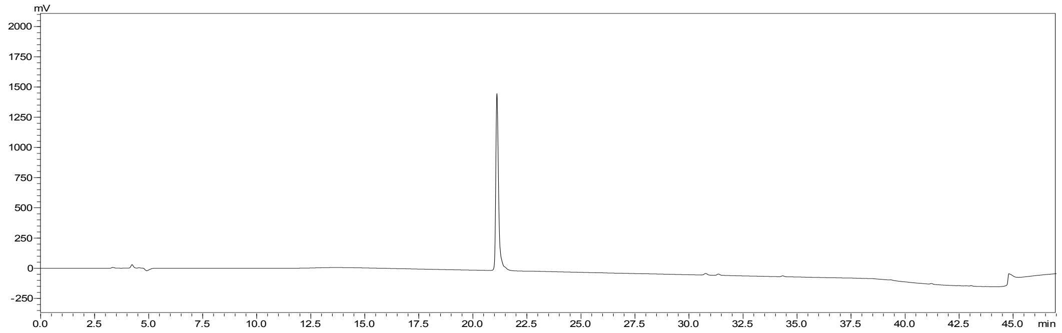

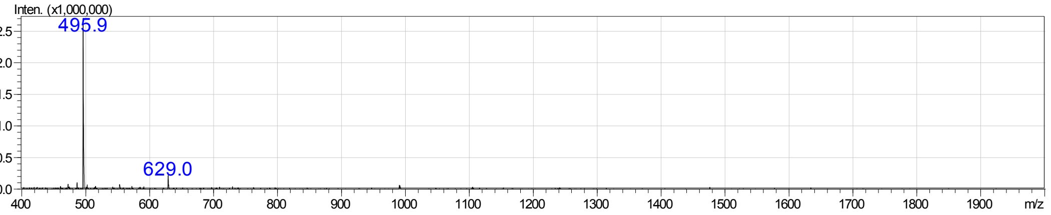

Ac-W-(cyclo-**i**)-MRGDM-NH_2 (**1i**)

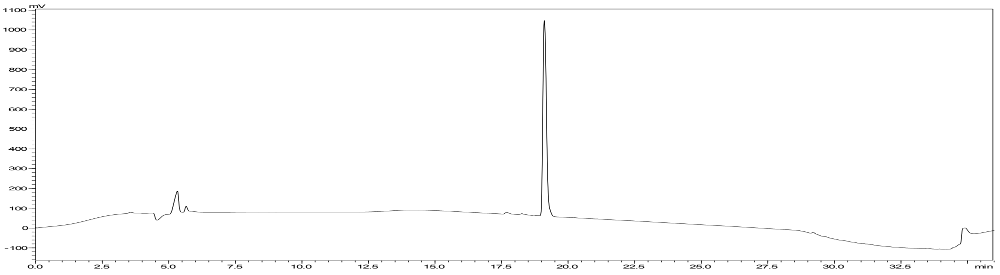

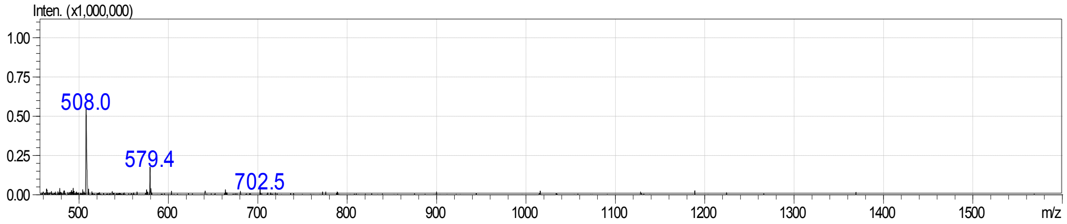

Ac-W-(cyclo-**c**)-MKIEMA-NH_2 (**2a**)

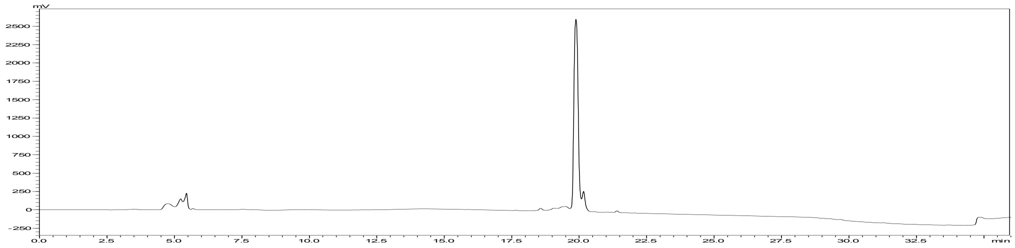

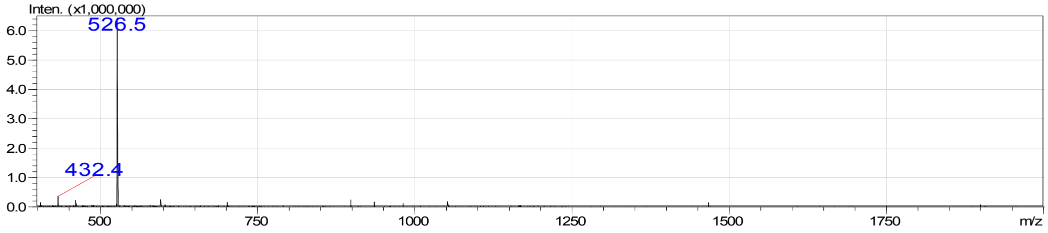

Ac-W-(cyclo-**c**)-MHVDMA-NH_2 (**3a**)

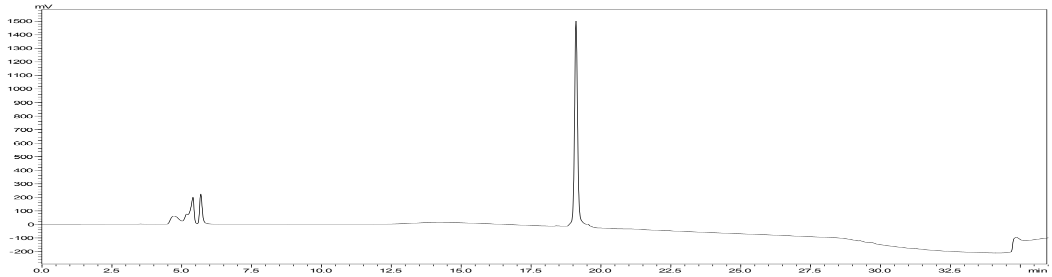

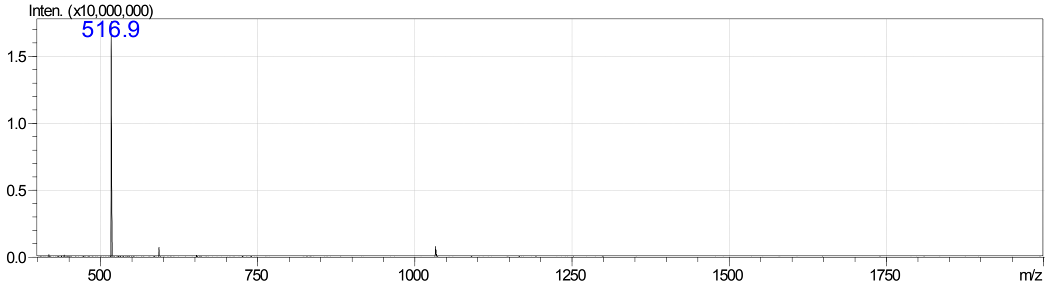

Ac-W-(cyclo-**c**)-MPYGMA-NH_2 (**4a**)

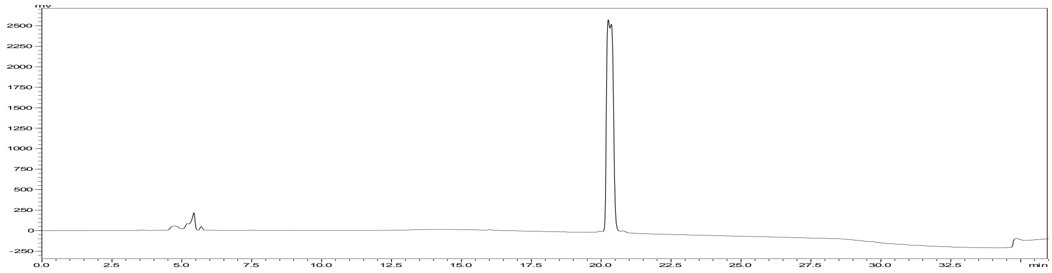

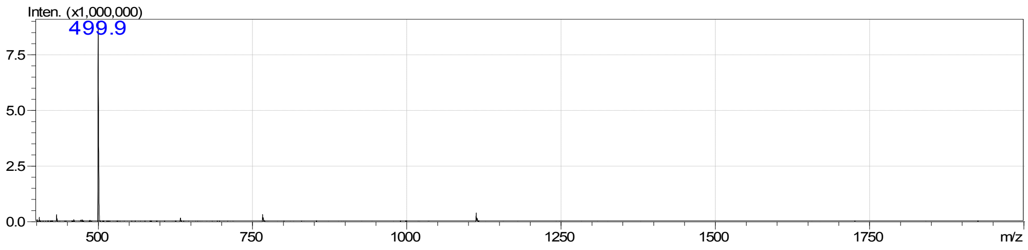

Ac-W-(cyclo-**c**)-MCLMA-NH_2 (**5a**)

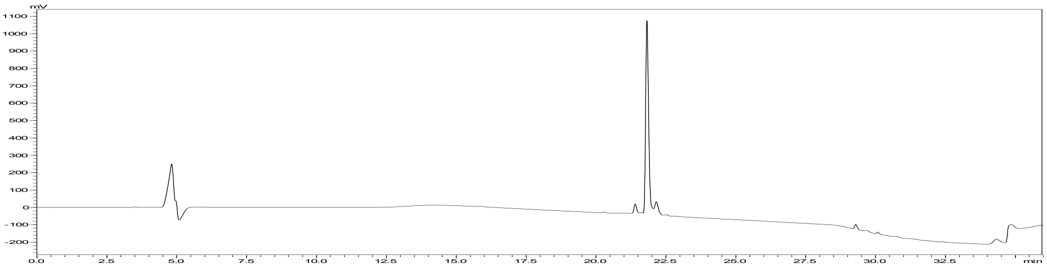

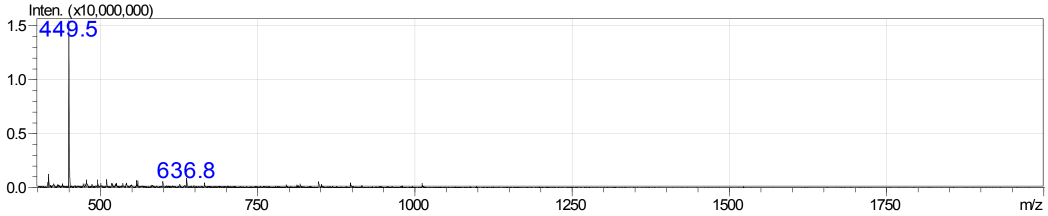

Ac-W-(cyclo-**a**)-MKEMA-NH_2 (**6a**)

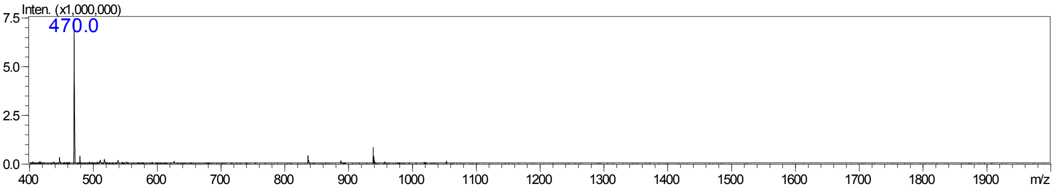

Ac-W-(cyclo-**a**)-MPHMA-NH_2 (**7a**)

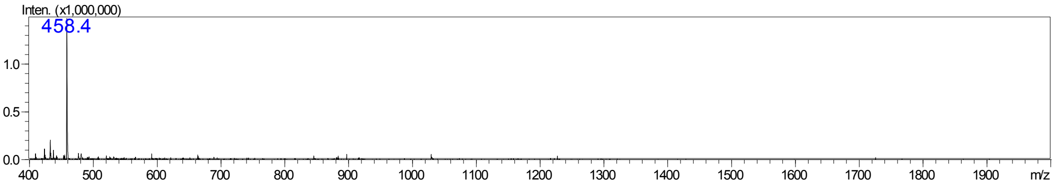

Ac-W-(cyclo-**c**)-MRGDRGDM-NH_2 (**8a**)

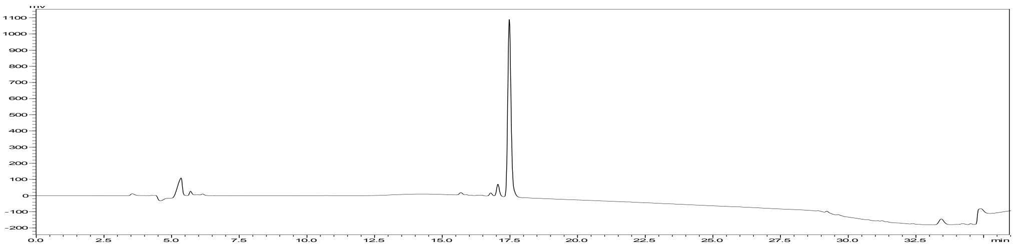

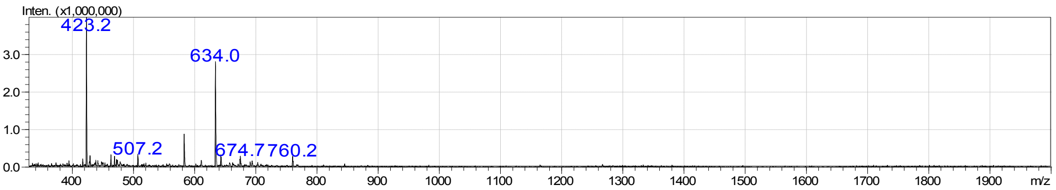

Ac-W-(cyclo-**c**)- βAMPQLPPMG-NH_2 (**9a**)

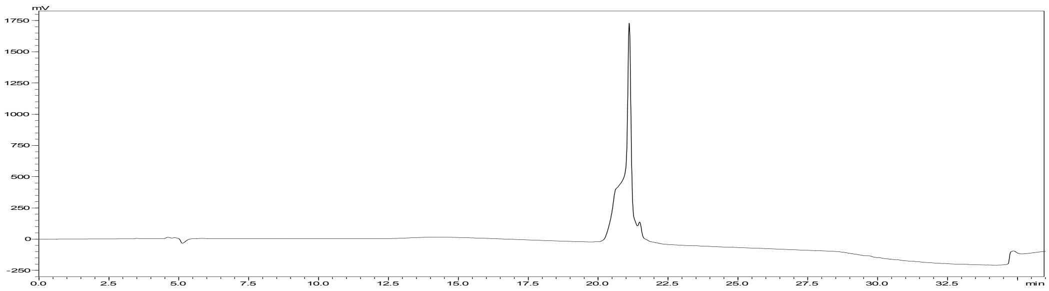

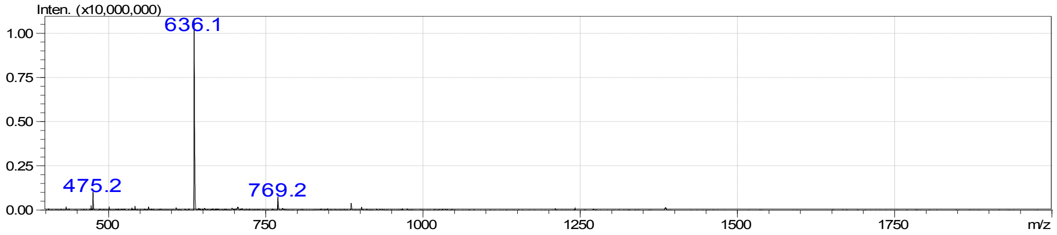

Ac-W-(bicyclo)-βAMHSRMPQLPPMG-NH_2 (**10j**)

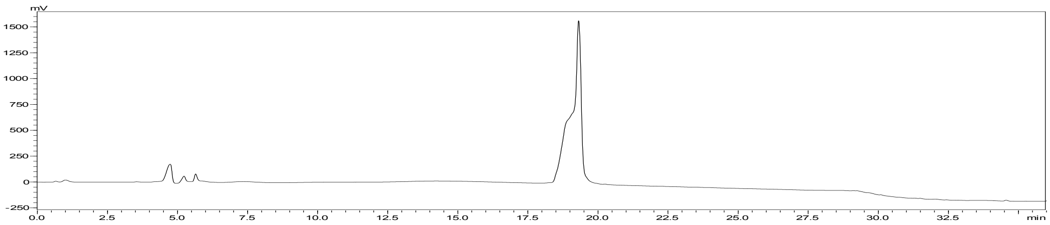

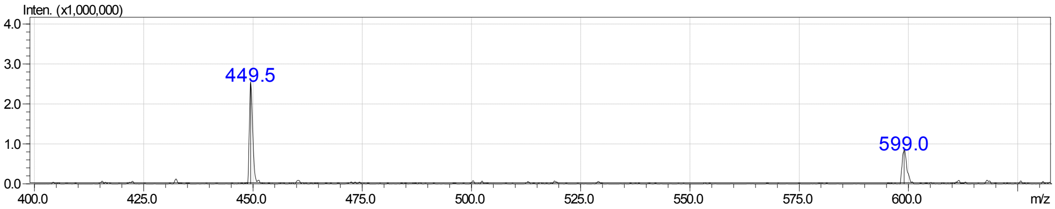

FITC-βA-MRRRM-NH_2 (**11**)

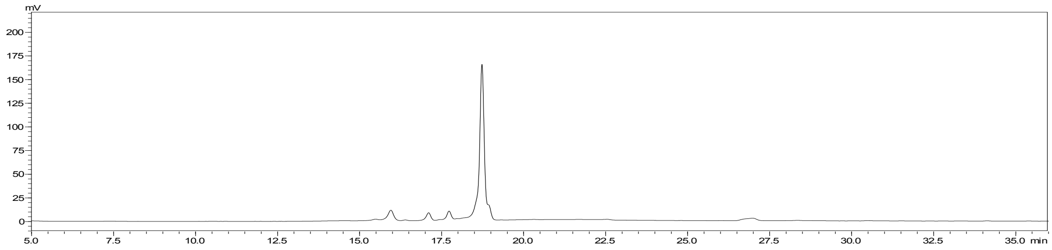

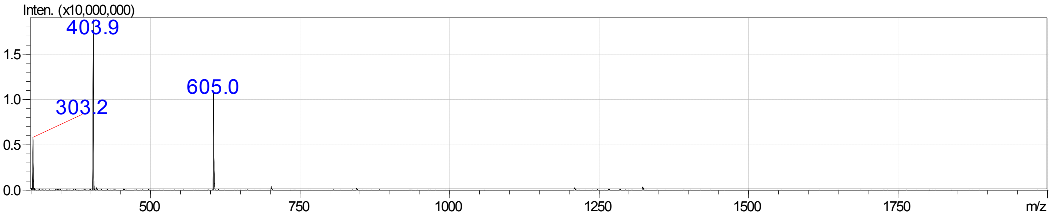

FITC-βA-(cyclo-**a**)-MRRRM-NH_2 (**11a**)

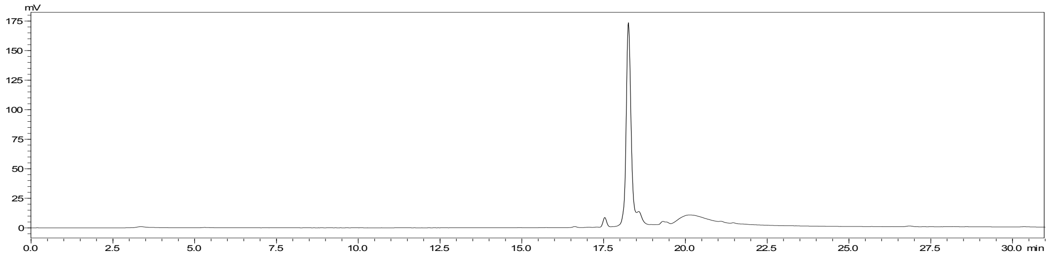

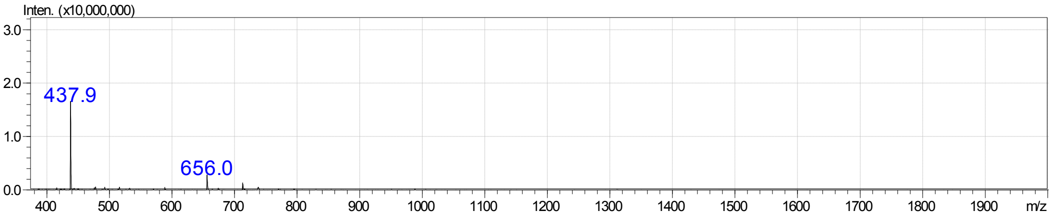

FITC-βA-(cyclo-**b**)-MRRRM-NH_2 (**11b**)

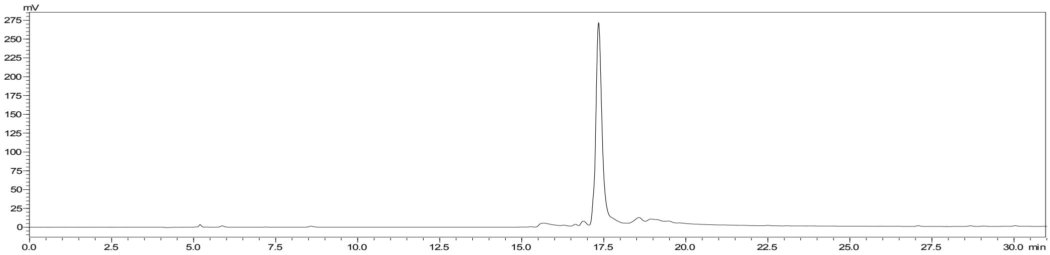

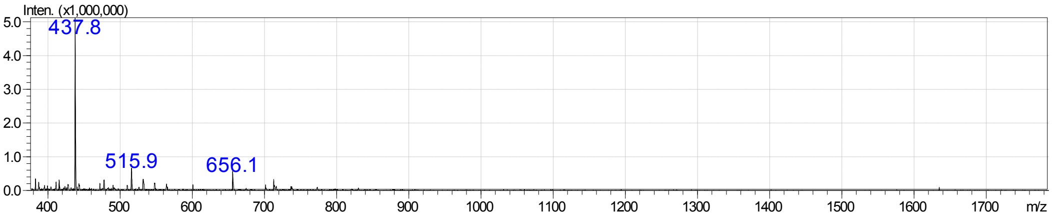

FITC-βA-(cyclo-**c**)-MRRRM-NH_2 (**11c**)

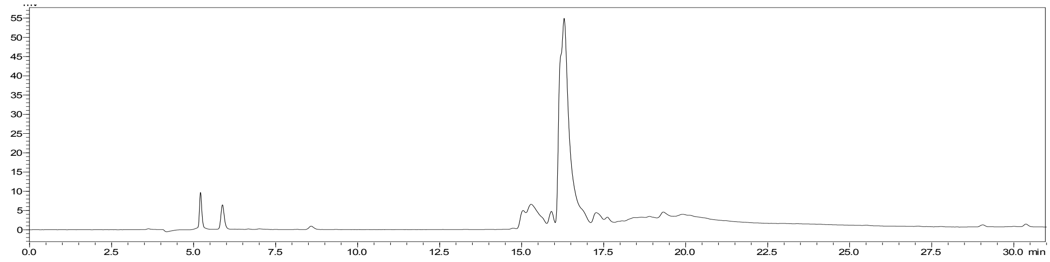

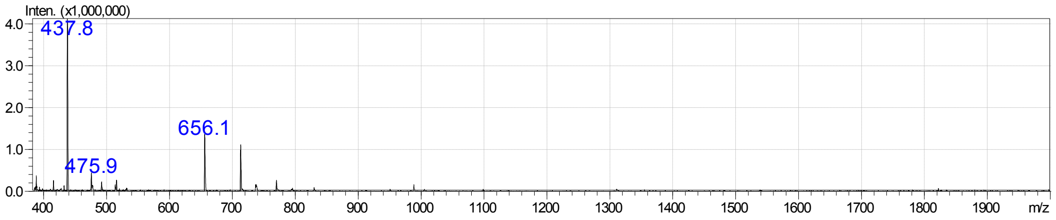

FITC-βA-(cyclo-**d**)-MRRRM-NH_2 (**11d**)

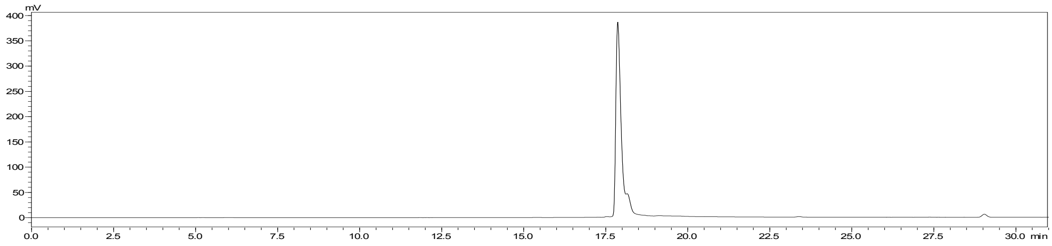

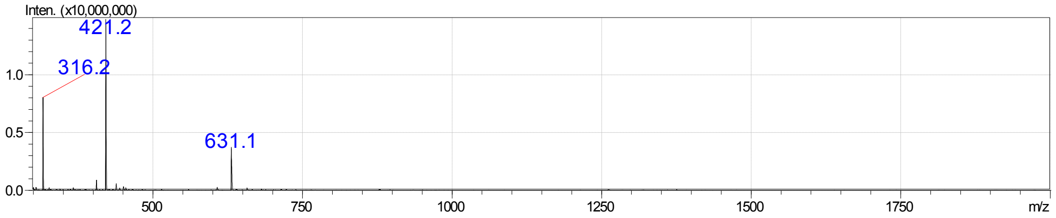

FITC-βA-(cyclo-**e**)-MRRRM-NH_2 (**11e**)

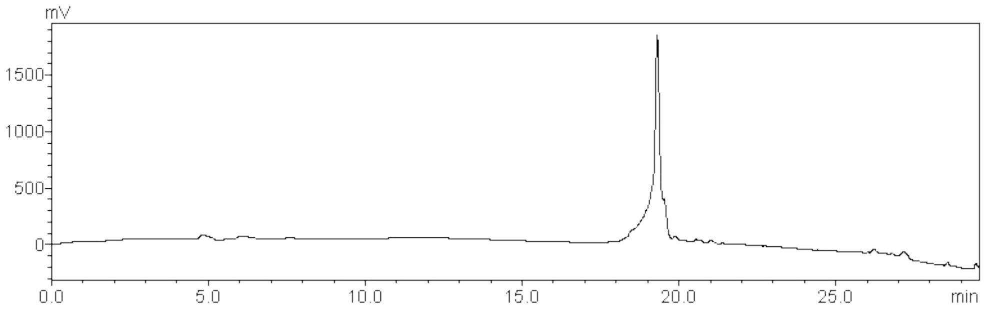

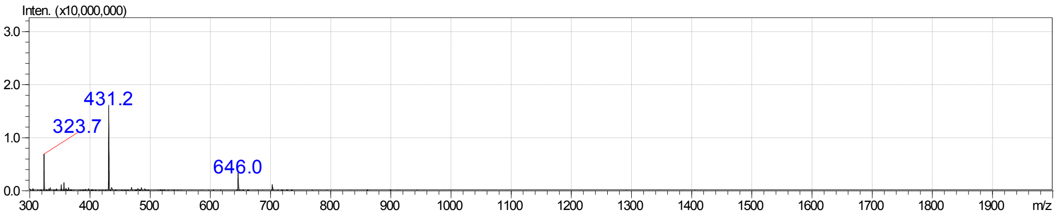

FITC-βA-(cyclo-**h**)-MRRRM-NH_2 (**11h**)

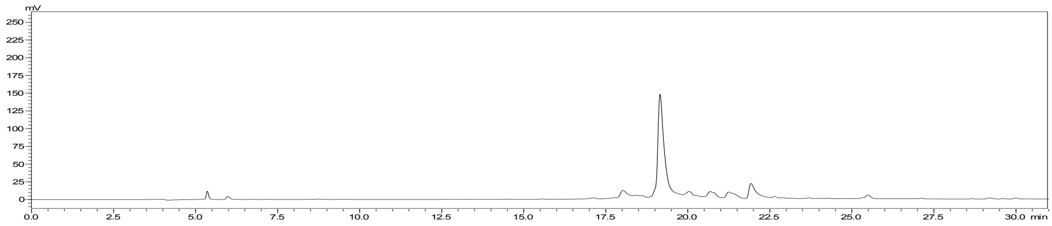

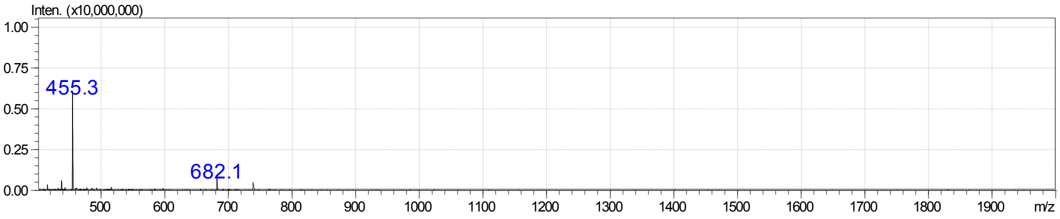

FITC-βA-(cyclo-**i**)-MRRRM-NH_2 (**11i**)

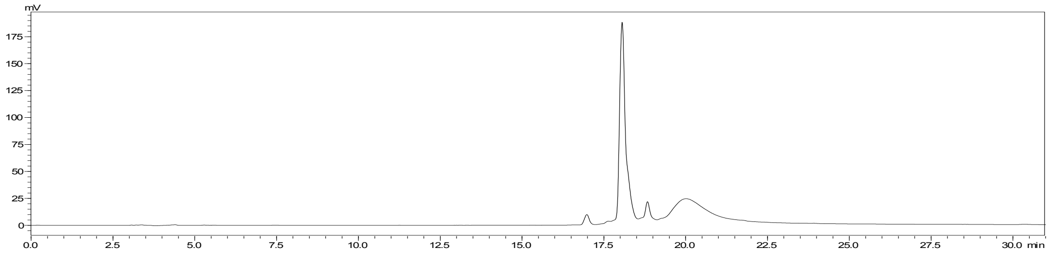

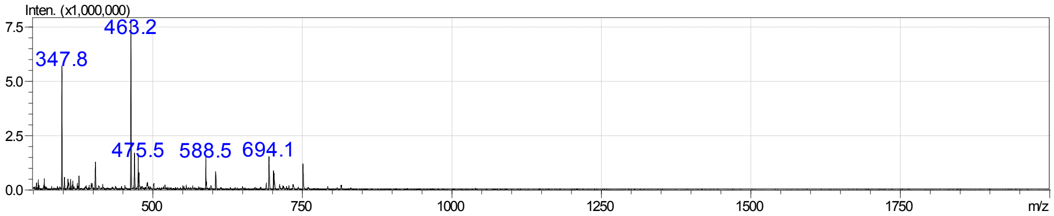

FITC-βA-(cyclo-**c**)-CRRRC-NH_2 (**12c**)

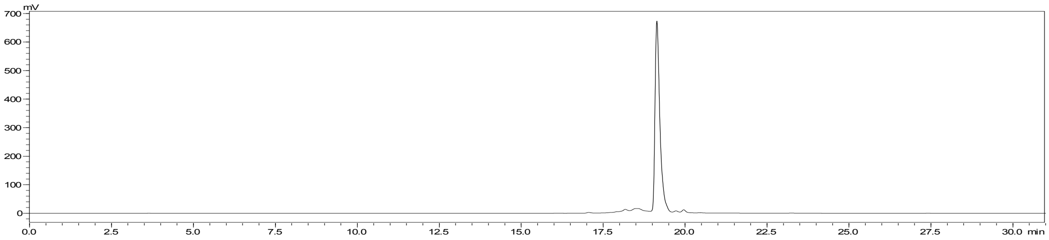

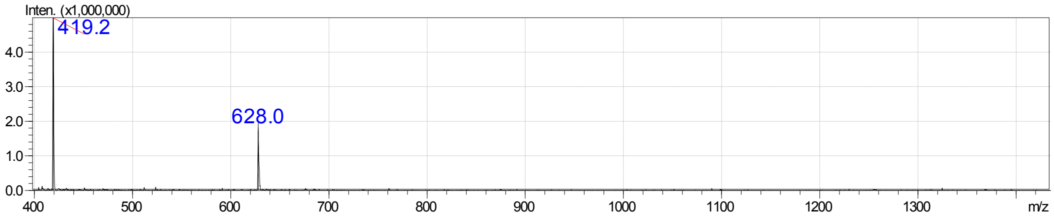

FITC-βA-(cyclo-**c**)- hCRRRhC-NH_2 (**13c**)

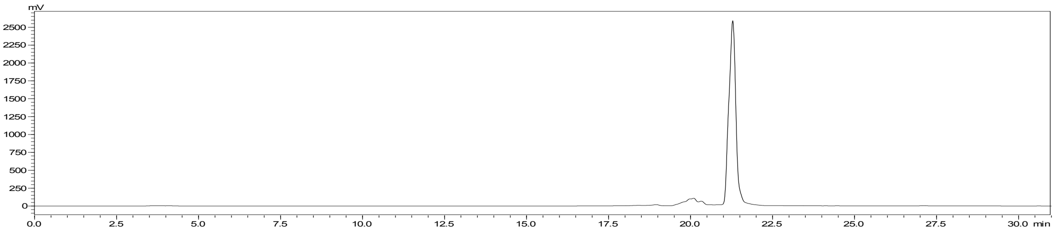

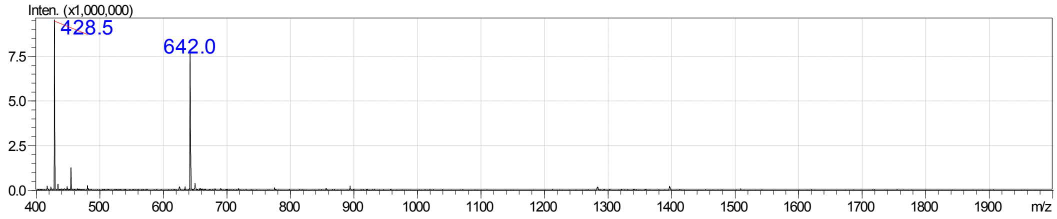

FITC-βA-RKKRRQRRR-NH_2 (**TAT**)

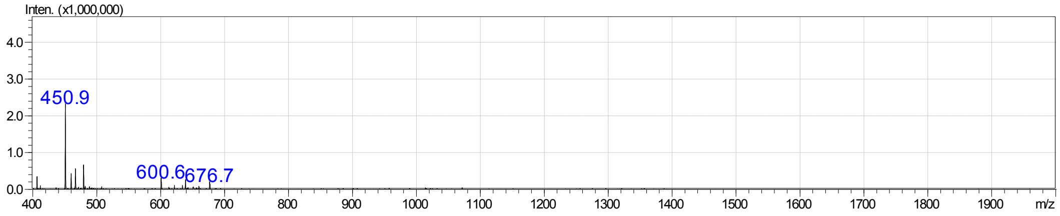

FAM-βA-RMILMRLLQ (**14**)

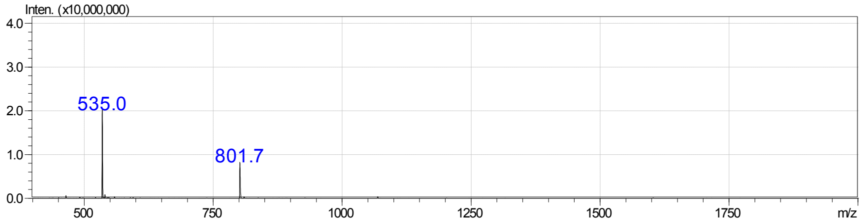

FAM-βA-(cyclo-**c**)-RMILMRLLQ (**14c**)

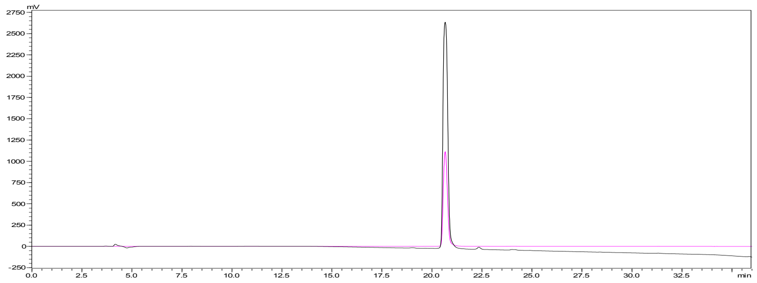

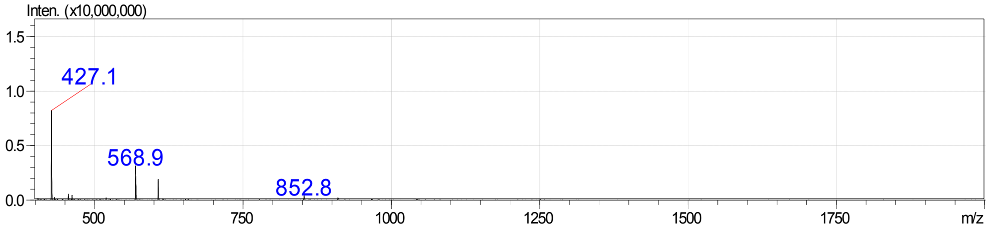

FAM-βA-MCNVVPLY(po3)DLLLEM (**15**)

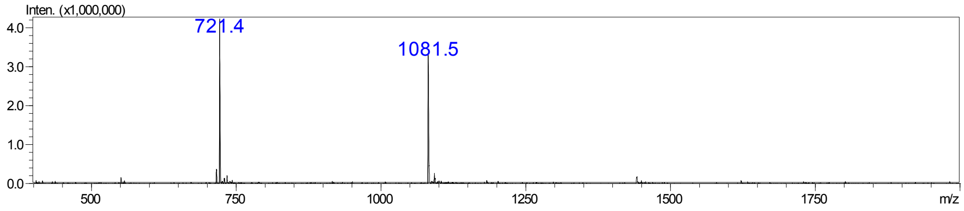

FAM-βA-(cyclo-**c**)--MCNVVPLY(po3)DLLLEM (**15c**)

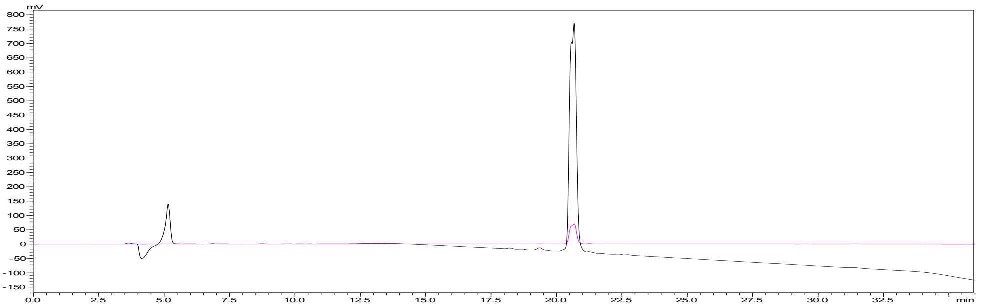

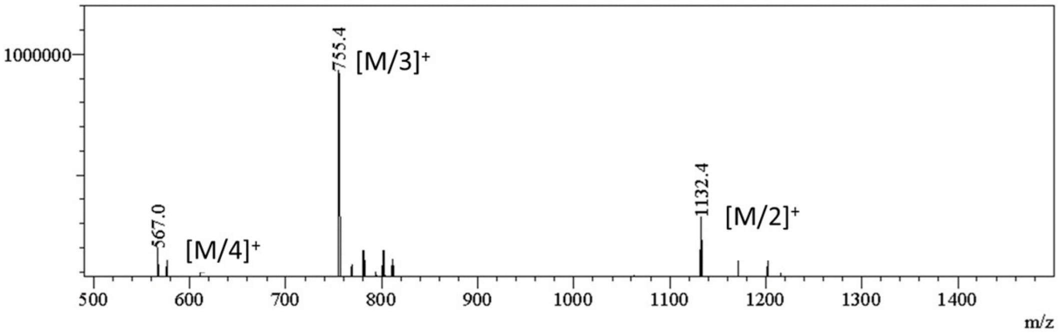

cyclo-**i**-Met-Asp-Nle-Met-Asp-AMC (**17i**)

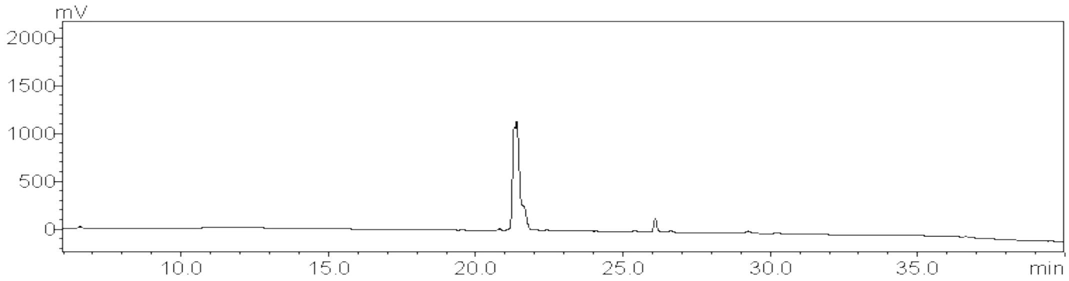

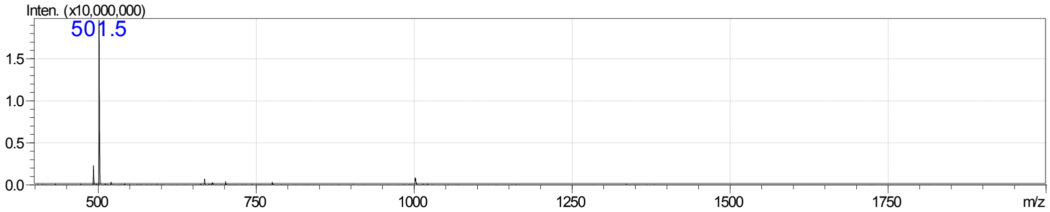

cyclo-**i**-Cys-Asp-Nle-cys(d)-Asp-AMC (**18i**)

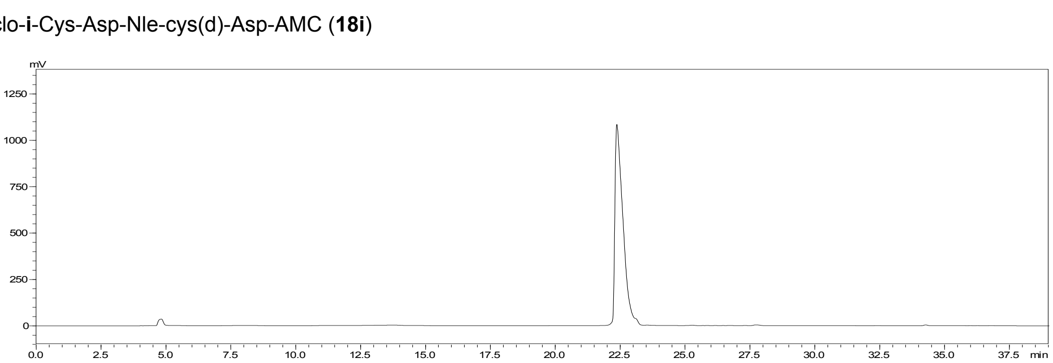

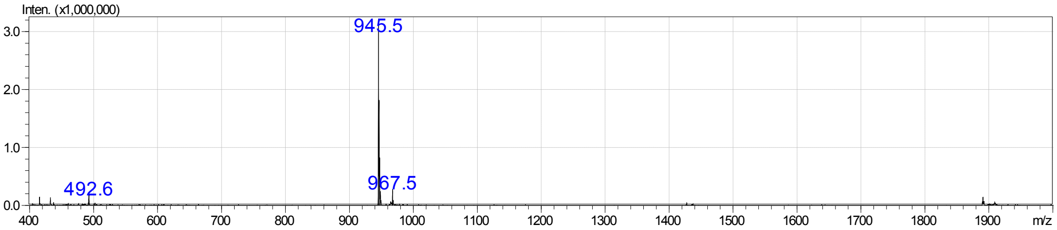

Reference

1. Jessica R. Kramer, Timothy J. Deming. Reversible chemoselective tagging and functionalization of methionine containing peptides. *Chem. Commun.*, **2013**, 49, 5144-5146.

2. Ziqing Qian, Xiaohua Xu, Jeanine F. Amacher, Dean R. Madden, Estelle Cormet-Boyaka, Dehua Pei. Intracellular Delivery of Peptidyl Ligands by Reversible Cyclization: Discovery of a PDZ Domain Inhibitor that Rescues CFTR Activity. *Angew. Chem. Int. Ed.*, **2015**, 54, 5874 –5878.
